# Cross‐scale regulation of seasonal microclimate by vegetation and snow in the Arctic tundra

**DOI:** 10.1111/gcb.16426

**Published:** 2022-09-24

**Authors:** Jonathan von Oppen, Jakob J. Assmann, Anne D. Bjorkman, Urs A. Treier, Bo Elberling, Jacob Nabe‐Nielsen, Signe Normand

**Affiliations:** ^1^ Section for Ecoinformatics & Biodiversity, Department of Biology Aarhus University Aarhus C Denmark; ^2^ Center for Biodiversity Dynamics in a Changing World (BIOCHANGE), Department of Biology Aarhus University Aarhus C Denmark; ^3^ Department of Biological and Environmental Sciences University of Gothenburg Gothenburg Sweden; ^4^ Gothenburg Global Biodiversity Centre Gothenburg Sweden; ^5^ Arctic Research Centre, Department of Biology Aarhus University Aarhus C Denmark; ^6^ Center for Permafrost (CENPERM), Department of Geosciences and Natural Resource Management University of Copenhagen Copenhagen Denmark; ^7^ Department of Ecoscience Aarhus University Roskilde Denmark

**Keywords:** Arctic tundra, microclimate, plant functional types, shrub expansion, snow cover, soil temperature, stratified random sampling, temperature offset

## Abstract

Climate warming is inducing widespread vegetation changes in Arctic tundra ecosystems, with the potential to alter carbon and nutrient dynamics between vegetation and soils. Yet, we lack a detailed understanding of how variation in vegetation and topography influences fine‐scale temperatures (“microclimate”) that mediate these dynamics, and at what resolution vegetation needs to be sampled to capture these effects. We monitored microclimate at 90 plots across a tundra landscape in western Greenland. Our stratified random study design covered gradients of topography and vegetation, while nested plots (0.8–100 m^2^) enabled comparison across different sampling resolutions. We used Bayesian mixed‐effect models to quantify the direct influence of plot‐level topography, moisture and vegetation on soil, near‐surface and canopy‐level temperatures (−6, 2, and 15 cm). During the growing season, colder soils were predicted by shrub cover (−0.24°C per 10% increase), bryophyte cover (−0.35°C per 10% increase), and vegetation height (−0.17°C per 1 cm increase). The same three factors also predicted the magnitude of differences between soil and above‐ground temperatures, indicating warmer soils at low cover/height, but colder soils under closed/taller canopies. These findings were consistent across plot sizes, suggesting that spatial predictions of microclimate may be possible at the operational scales of satellite products. During winter, snow cover (+0.75°C per 10 snow‐covered days) was the key predictor of soil microclimate. Topography and moisture explained little variation in the measured temperatures. Our results not only underline the close connection of vegetation and snow with microclimate in the Arctic tundra but also point to the need for more studies disentangling their complex interplay across tundra environments and seasons. Future shifts in vegetation cover and height will likely mediate the impact of atmospheric warming on the tundra soil environment, with potential implications for below‐ground organisms and ecosystem functioning.

## Graphical abstract

Across an Arctic tundra landscape, vertical temperature differences between above‐ and below‐ground systems were greater with denser and taller shrub‐ and bryophyte‐dominated vegetation (during summer) and longer snow cover duration (during winter). In our stratified random study, these effects exceeded relationships with topography, highlighting the importance of vegetation and snow in controlling tundra microclimate. As our findings were consistent across sampling scales, we outline how these results could facilitate integration of remote sensing data with locally measured cover and temperature values.

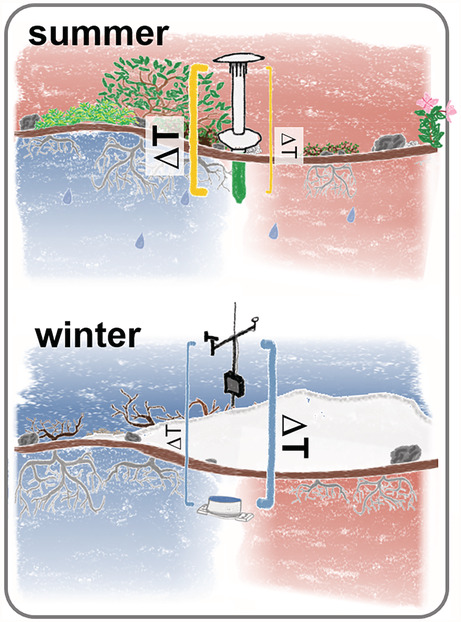

## 1 INTRODUCTION

The Arctic is experiencing climate change at unparalleled magnitudes, as air temperatures have been increasing at rates at least three times the global average and precipitation has increased by more than 9% over the last 50 years (AMAP, 2021; Rantanen et al., 2022). These trends have induced rapid change in tundra ecosystems across the Arctic, such as altered plant species composition (Elmendorf et al., 2015) and increased canopy height in plant communities (Bjorkman et al., 2018). Shrub cover has increased in many Arctic tundra regions (García Criado et al., 2020; Myers‐Smith et al., 2011; Tape et al., 2006) with potentially important feedback to the soil environment (Kemppinen et al., 2021; Myers‐Smith & Hik, 2013) and permafrost dynamics (Blok et al., 2010; Heijmans et al., 2022). However, while most biological processes in short‐statured tundra vegetation take place close to the ground or in the shallow soil layer, temperatures are commonly monitored by climate stations at 2 m height. These temperature measurements provide an important macroclimatic baseline, yet they likely fail to capture conditions relevant for most tundra organisms (sensu Lembrechts et al., 2020, 2022). To accurately assess and predict tundra ecosystem processes including vegetation development for organisms at different heights, it is therefore important to determine how site‐specific factors affect free‐air, canopy‐level, near‐surface, and soil temperature, as well as the temperature difference among these layers (Convey et al., 2018).

Local vegetation and topography can alter environmental conditions above, near, and below the soil surface (Aalto et al., 2018; Bramer et al., 2018; Geiger, 1965; Lenoir et al., 2013). In the tundra, shading from standing vegetation dominated by shrubs can reduce soil temperatures and soil temperature fluctuations during the growing season (Aguirre et al., 2021; Blok et al., 2010; Kade et al., 2006; Klene et al., 2001; Myers‐Smith & Hik, 2013). Cooling of soils has also been observed under insulating mats of bryophytes (Blok et al., 2011; Gornall et al., 2007; van der Wal & Brooker, 2004) or lichens (Cannone & Guglielmin, 2009; Mallen‐Cooper et al., 2021; van Zuijlen et al., 2020). Furthermore, soils are commonly colder in depressions or shady locations within topographically heterogeneous landscapes (Aalto et al., 2018; Opedal et al., 2015), and particularly where high soil moisture induces evaporational cooling (Aalto et al., 2013). During winter and early spring, insulation from snow cover, which accumulates in dense shrub vegetation or lee positions (Sturm et al., 2001), leads to soils that are warmer than above‐ground layers (Aalto et al., 2018; Kade et al., 2006). This effect can even outweigh summer cooling and result in a net annual warming of soils under tall shrub canopies (Kropp et al., 2021). However, tall shrubs can also reduce snow insulation of soils in spring, as dark branches penetrating the snow increase the radiative heat input and accelerate snow melt (Wilcox et al., 2019).

Through these effects on soil temperature, vegetation and topography can influence soil microbial community composition (Zak & Kling, 2006), nutrient cycling (Gornall et al., 2007; Mueller et al., 1999), and ecosystem fluxes (Cahoon et al., 2012; Lafleur & Humphreys, 2018; Shaver et al., 2006; Sturm et al., 2001). In addition, microclimatic variation above and below the soil surface across tundra vegetation types can affect abundance of organisms from higher trophic levels such as arthropods (Høye et al., 2021). To accurately assess and predict these effects, we need precise measures of temperatures throughout the vegetation profile, matching activity zones of different organisms (i.e., above‐ and below‐ground microclimate) and accounting for the influence of vegetation, topography, soil moisture, and snow on temperature (Lembrechts et al., 2020).

Ecological relationships can vary considerably with the scale studied, that is, spatial resolution and extent (Wiens, 1989). To gain a detailed understanding of how vegetation modifies macroclimate and hence microclimatic variation in space (Lembrechts et al., 2019; Lenoir et al., 2013), it is important to study the relationship at different spatial resolutions and cover the entire spectrum of spatial variation within a study site. Most previous studies of thermal differences between layers of tundra vegetation have investigated predefined, subjectively selected vegetation types (Kade et al., 2006; Mallen‐Cooper et al., 2021; but see Aalto et al., 2018), without considering vegetation–microclimate relationships across different spatial sampling resolutions. Yet, the effect of proximal vegetation (= fine‐resolution data) versus more distant vegetation (= coarse‐resolution data) for local microclimatic conditions in arctic tundra remains unknown. The scale dependency of ecological relationships also largely defines the necessary sampling effort, as larger areas are increasingly laborious to sample representatively. Identifying the optimal sampling resolution that offers a manageable compromise between effort and extent covered remains challenging, especially in remote Arctic locations. We could reduce such constraints if we achieved high predictability of microclimates from coarse‐resolution vegetation data, for instance derived from remotely sensed vegetation indices. However, to our knowledge, it is uncertain to what extent coarse‐grain vegetation data contribute to infer microclimate from regional macroclimate. Determining how far vegetation relationships with microclimate extend can therefore help to identify potential ways toward a more efficient monitoring and predictions of tundra microclimates.

**FIGURE 1:**
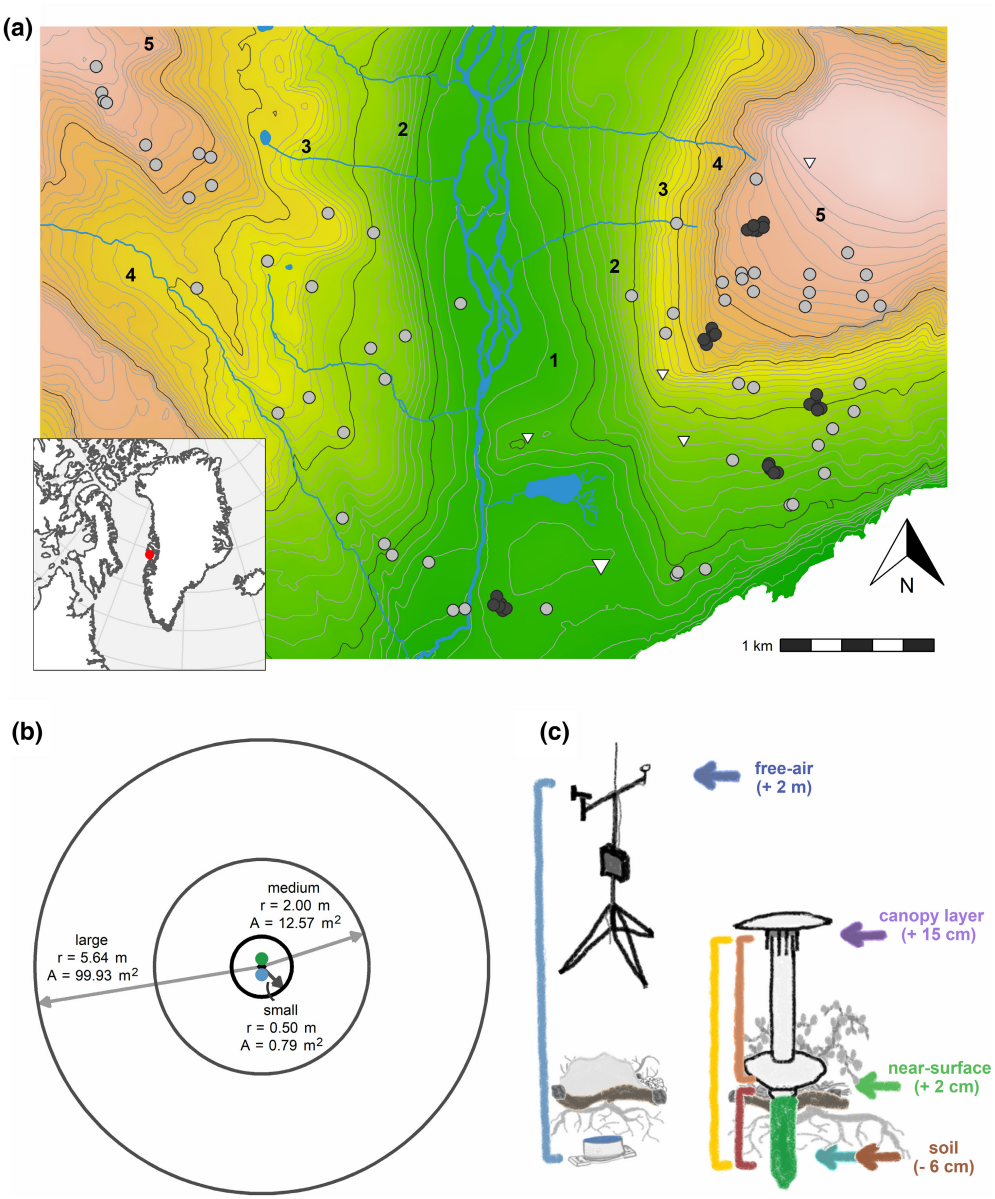
The locations and design of the microclimate monitoring. (a) We placed 90 plots across an arctic tundra landscape at Qeqertarsuaq (Disko Island), Western Greenland. We placed sample plots according to a stratified random design to capture variation in vegetation productivity, water availability, and elevation (dots). Sample plots within core areas are shown as black dots. Positions of weather stations are shown as triangles, and the enlarged triangle marks the lowest elevation station that was used as a free‐air temperature reference variable in our microclimate models. Background color indicates elevation (green low, brown/white high) and contour lines intervals of 20 m. The five elevation zones are delimited with black lines. (b) At each sample plot, we placed a TOMST TMS‐4 logger (green dot) 10 cm north and a HOBO logger (blue dot) 10 cm south of the plot center. We monitored vegetation cover in three nested circles of different areas around the center (main analysis using data from small circles). (c) Local and microclimate were monitored with different sensors: TMS loggers measured temperature at three heights as well as soil moisture (Wild et al., 2019) across the growing season, HOBO loggers measured soil temperature during a complete year, and weather stations provided local air temperature. Vertical bars indicate sensor pairs for calculation of temperature differences. Note that plot locations (e.g., core area at elevation band 3) may fall into different elevation bands due to local topographic or substrate conditions (see text for details). Circles and loggers are not to scale.

We aimed to assess how microclimate is related to characteristics of the vegetation relative to macroclimate, topography, soil moisture, and snow, across a below‐ and above‐ground vertical vegetation profile (−6 to 15 cm) and at different spatial resolution (0.8–100 m^2^) in the Arctic tundra. We recorded temperatures at three different heights during the growing season, representing different vegetation layers, and in the soil during winter, as well as vegetation, soil moisture, and topographical parameters in nested plots of different sizes. In addition, we compared in situ temperatures with macroclimate from both seasons obtained as free‐air records from nearby weather stations. We placed 90 plots stratified randomly across a mountainous tundra landscape on Qeqertarsuaq (Disko Island), Western Greenland (Figure 1a), to cover the available environmental gradients of topography, moisture conditions, and vegetation types. We calculated seasonal averages of daily mean, minimum, and maximum temperatures for each of the vegetation layers, as well as vertical temperature differences between them, to test the following hypotheses:

1. During the growing season, higher shrub and bryophyte cover and a taller canopy reduce near‐surface and soil temperature, but have a minor effect on canopy‐level temperature.
2. During winter, higher shrub cover and taller vegetation extend snow cover duration, resulting in warmer soils compared to free‐air conditions.
3. The power of vegetation cover to predict temperature differences at the plot center decreases with increasing scale of the vegetation cover measurements (i.e., plot size).

The outcomes of this study will improve our understanding of which biotic and abiotic factors drive tundra microclimate and whether coarse‐grained predictions from climate models also represent local processes. It will therefore provide important insights into how future vegetation changes could affect temperature‐sensitive ecosystem processes in the Arctic tundra.

## 2 METHODS

### 2.1 Study area

All study sites were situated along the eastern and western slope of Blæsedalen valley near Qeqertarsuaq (Godhavn), Disko Island, Greenland (69°16′N, 53°28′W, bioclimatic subzone D), spanning an area of 15 km^2^ of mountainous and topographically heterogeneous tundra (Figure 1a). Raynolds et al. (2019) classified the vegetation as prostrate to erect dwarf shrub tundra (P1, P2, S1). Throughout the valley and the slopes, dense vegetation of low, erect shrubs (e.g., *Betula nana, Empetrum hermaphroditum, Salix glauca, Vaccinium uliginosum*), with herbaceous plants, mosses, and lichens in the understory dominates the landscape. On the flat mountaintops, more patchy prostrate shrubs (mainly *S. glauca*) and bog vegetation dominate, possibly due to the presence of meltwater from long‐lasting snow patches. Underlain by discontinuous permafrost, soil substrates across the study area are generally well drained and, somewhat differing from other Arctic locations, dark, basaltic, and Fe‐rich (Xu et al., 2021). Maximum active layer thickness varies from about 0.4 m in wet depressions to more than 2 m in drier areas and at higher altitudes (Rasmussen et al., 2022).

### 2.2 Stratified random placement of sample plots and microclimate monitoring

Prior to the field campaign, we divided the study area into five elevation bands of equal altitudinal range (160 m each), based on the ArcticDEM digital surface model (Porter et al., 2018), to capture variation of temperature and vegetation across elevation. Furthermore, we classified the study area into three greenness classes based on the Normalized Difference Vegetation Index (NDVI) as a proxy for productivity and into areas of high and low wetness based on the Normalized Difference Wetness Index (NDWI; Gao, 1996) as a proxy for water availability to plants (Table S1). Both NDVI and NDWI were generated from Sentinel‐2 Multispectral Imagery (European Space Agency, 2015) derived from a single cloud‐free scene taken on July 30, 2018. From both layers, we masked out areas with (i) a slope angle of more than 30°, to allow for safe access; (ii) water bodies (NDWI ≤ 0.3); and (iii) snow (Normalized Difference Snow Index ≤ 0; Dozier, 1989). We then combined the two layers with the elevation bands as the basis for our stratified (vegetation greenness, moisture, and altitude) random placement of sample plots (see Supplementary Note for an assessment of the local conditions in relation to the intended classes).

We placed 90 plots across the landscape, of which 60 were scattered across both mountain slopes and 30 were located in clustered “core areas” on the eastern slope (Figure 1a; see Supplementary Note for details). At 10 cm distance from the plot center toward true North, we placed a TMS microclimate logger (TMS‐4; TOMST; Wild et al., 2019) (Figure 1b). These loggers conduct parallel temperature measurements at 6 cm below ground (hereafter: soil temperature) and at 2 cm (near‐surface temperature) and 15 cm (in the following considered canopy‐level temperature) above ground. The TMS loggers also record moisture in the topsoil (to approx. 14 cm depth; Figure 1c). The position of the temperature sensors might vary slightly due to local soil conditions (Figure S1). In addition, at 10 cm distance toward true south from each plot center, we installed a HOBO data logger (MX‐2201; Onset Computer Corp.) at 6 cm below ground (Figure 1b,c).

We placed all loggers between June 26 and July 1, 2019. The TMS loggers were retrieved between 8 and 12 August 2019 while the HOBO loggers were left for ~14 months and retrieved between 19 and 27 August 2020. We set TMS loggers to record temperature and soil moisture at 10‐min intervals while HOBO loggers recorded temperature at 15‐min intervals to increase battery longevity. Whenever possible throughout the season, we checked for correct positioning and intactness of TMS loggers and their radiation shields.

### 2.3 Vegetation and topographic variables

We conducted vegetation surveys in nested circular sample plots with areas of 0.79 (small), 12.57 (medium), and 99.93 m^2^ (large), respectively, to obtain data at different resolutions (Bøcher, 1935; Figure 1b), between July 2 and August 12, 2019. The area covered by the large sample plots corresponds to the 100 m^2^ resolution of WorldView and Sentinel‐2 imagery. Starting from the small to the large circle, we recorded the cover of shrubs, forbs, graminoids, bryophytes, lichens, solid rock/stones (diameter *d* > 10 cm), gravel/bare ground (*d* < 10 cm), and litter for each sample plot. Standing on opposite sides of each plot, each of two observers gave an independent estimate before agreeing on a cover value. We assessed two‐dimensional cover of each functional type visually in the following intervals: <5%, 5%–10%, 10%–20%, 20%–30%, continuing in 10% intervals until 90%–100% thereafter. Prior to analysis, we set cover values to the mean of each interval (i.e., 2.5%, 7.5%, 15%, 25%, etc.). However, we did not incorporate forbs and graminoids into our analyses because of their generally scarce cover compared to other functional types (Figure S2). As we assessed cover of each functional type separately, cumulative vegetation cover in any one plot could exceed 100% in case of overlapping vegetation. At time of logger collection, we also recorded the percentage of vegetation cover directly at the height of each temperature sensor, that is, looking down at the logger from vertically above and estimating the cover at the respective height for a circle with a radius of 5 cm. For lower TMS sensors, we removed reflection shields to ensure clear vision from above. In addition, we assessed vegetation height by lowering a horizontal measuring stick into the vegetation until an estimated half of its surface was covered by vegetation. We repeated this procedure four times at randomly chosen locations within each plot and subsequently averaged measurements.

We also measured inclination and orientation of the slope across the 4‐m circle diameter at each plot using a hand‐held clinometer. Based on locally measured slope inclination and orientation, as well as local solar zenith angle and declination, we calculated incoming solar radiation as the mean Solar Radiation Index (SRI; Keating et al., 2007) across the growing season or winter logging period (see Section 2.6). In addition, we classified the topographic position (“landform”) of each plot on a five‐step scale from sheltered to exposed locations (snowbed/depression/flat/slope/ridge).

### 2.4 Soil moisture and snow cover variables

We collected soil samples in each plot, which we analyzed for fractions of sand, silt, and clay. We then used these data to assign each plot a predefined soil type as provided by Wild et al. (2019) based on minimum Euclidean distance for particle size fractions, and converted raw soil moisture measurements from the TMS loggers using a second‐degree polynomial with soil type‐specific coefficients (see appendix A in Wild et al., 2019). We inspected each soil moisture time series visually and removed records for specific periods during which below‐ground logger parts had been exposed, which were identified from soil temperature records (see below). As calibration yielded a few negative values for some sensors in rocky soil, we scaled all soil moisture values between 0 and 1 to conserve relative differences between plots. In addition, we completely excluded moisture time series that did not show a marked difference from a value measured in air for the logging period, perhaps due to insufficient contact of the sensor with the soil matrix (*n* = 2). We then calculated mean soil moisture over the growing season for each of the remaining 88 plots. To assess its representativeness for moisture extremes during the growing season, we correlated seasonal mean values to lower and upper 90% quantiles of individual soil moisture measurements. Seasonal mean values were highly correlated with both quantiles (lower 90%: Pearson's *r* = .99; upper 90%: *r =* .86), indicating that variation in mean soil moisture sufficiently represented soil moisture extremes.

Snow cover insulates the ground surface and soil and substantially reduces daily amplitudes in near‐surface and soil temperatures (Zhang et al., 1997). Therefore, we counted the number of days with a soil temperature amplitude of <2°C (Zhang et al., 1997) to derive the period of snow cover between November 1, 2019 and March 31, 2020 for each of the 83 plots with a complete winter soil temperature record (see below).

### 2.5 Productivity data for extrapolation tests

To test for relationships between microclimate and gridded productivity data as a proxy for vegetation biomass, we obtained a cloud‐free Sentinel‐2 scene of the study area (European Space Agency, 2015) from around peak growing season during our study period (July 29, 2019). We used bands three (red) and eight (near‐infrared) at 10 m resolution to calculate simplified kernel NDVI (kNDVI; Camps‐Valls et al., 2021), a generalization of NDVI which accounts for the nonlinear relationship of NDVI with vegetation biomass (Myers‐Smith et al., 2020). Finally, we extracted weighted kNDVI for the exact areas covered by our large sample plots.

### 2.6 Temperature response variables

#### 2.6.1 General calculation of temperature variables

**FIGURE 2:**
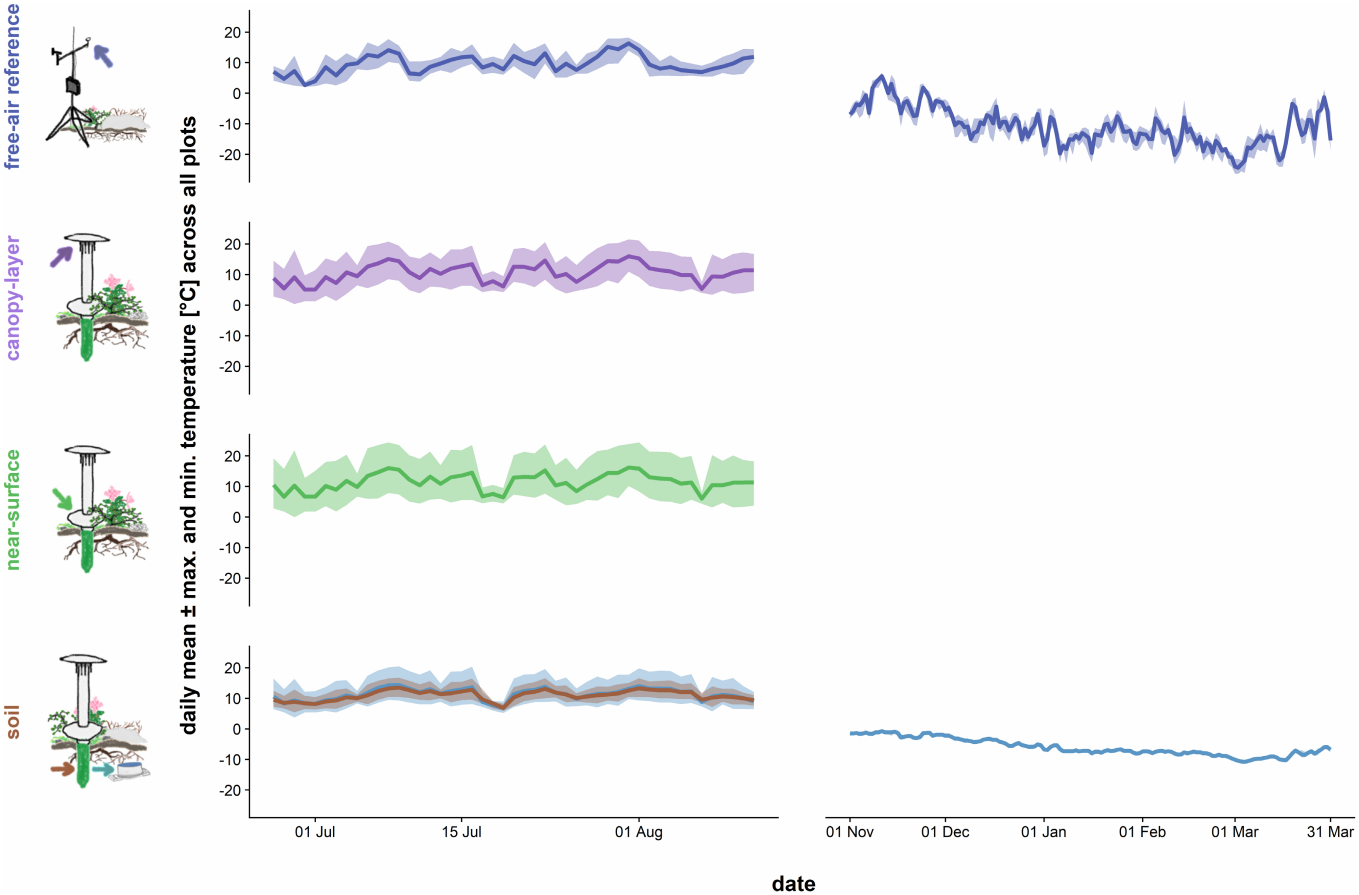
Daily temperature variation was smaller in the soil than for above‐ground layers, and larger during the growing season than in winter. Lines represent daily mean temperatures in free‐air, canopy‐height, near‐surface and soil layers, averaged across all sample plots, while ribbons mark average daily minimum and maximum temperatures. Values from both TMS and HOBO sensors are presented for growing season soil temperatures to enable comparison. Vertical axis ranges are aligned to make ranges comparable. Note that growing season and winter measurement periods differ in length.

Analyses focused on two periods: the 2019 *growing season* (i.e., individual logging period of TMS sensor in each plot), and the 2019–2020 *winter* months (November 1 to March 31) with a high probability of snow cover for the study area (Figure 2). We calculated daily mean, minimum, and maximum temperatures for every sensor and plot, as well as pairwise temperature differences (Δ*T*) in daily mean, minimum, and maximum temperatures between the different sensors by subtracting the value from a higher mounted sensor from the value from a lower mounted sensor (i.e., soil–canopy; near‐surface–canopy; soil–near‐surface; soil–free‐air). A positive Δ*T* indicates relatively warmer temperatures in the respective lower stratum, while a negative Δ*T* indicates colder temperatures in the lower stratum. In addition, to estimate ecological relevance of temperature measurements, we calculated growing degree days as the sum of daily mean temperatures above 0 and 5°C, as well as differences in growing degree days for each sensor, over the growing season. We also calculated freezing degree days and respective differences for free‐air and soil temperatures over the winter period. All degree‐day variables were highly correlated with their reference mean temperature measurements (Tables S2 and S3), suggesting interpretability of results based on temperatures and temperature differences in ecological contexts. Below we describe the handling of the temperature data from the different sources in more detail.

#### 2.6.2 Growing season canopy‐level, near‐surface, and soil temperatures

For the growing season temperature records from the TMS loggers (between June 26 and August 12, 2019), we only included temperature data between the day after installation and the day before retrieval of each respective logger and excluded all temperature time series with an error code (*n* = 4 for canopy‐level sensor, *n* = 0 for near‐surface and soil sensors). We also excluded measurements from periods (±1 day) with atypically high daily maximum soil temperature (>30°C, *n* = 1) or soil temperature amplitude (>15°C, *n* = 2), which coincided with observed removal of the sensors from the soil, presumably due to animal disturbance. In addition, we checked canopy‐level temperature curves during periods where radiation shields were removed, but did not detect any notable anomalies.

#### 2.6.3 Winter soil temperatures

For the soil temperature data from HOBO loggers, we likewise excluded measurements from periods during which loggers were exposed on the surface (±1 day; *n* = 4). In addition, we removed all soil temperature records from the HOBO loggers that we found exposed at the time of retrieval (*n* = 6) for all dates later than the date of the last check during TMS retrieval. We also removed one HOBO logger time series that had an unusually high number of days with maximum temperature >30°C. Across all plots, daily mean HOBO soil temperature records were overall consistent with parallel TMS soil temperatures during the growing season (Figure 2, Figure S3), indicating a high alignment of measurements for comparison across seasons. We therefore only used the HOBO soil temperature record from the winter period for analyses (see below).

#### 2.6.4 Free‐air temperatures from weather stations

Even though our TMS canopy‐level sensors actually reached above the low‐lying tundra vegetation in 87 of 90 plots (Figure S1), these measurements might still be subject to boundary layer effects from the vegetation (Geiger, 1965). In addition, limited hardiness of the standardized TMS logger shields prevented us from leaving TMS loggers out to monitor canopy‐level temperatures throughout winter. To assess the comparability of canopy‐level and free‐air temperatures, and to be able to compare temperatures above and below the snow cover and vegetation during winter, we retrieved air temperatures from five permanent weather stations across the study area (Figure 1a,c). These stations are being maintained by the Greenland Ecosystem Monitoring programme (https://g‐e‐m.dk/) and record temperatures at 30‐min intervals. We removed all measurements with a reported error before calculating daily mean, minimum, and maximum temperatures. We determined which weather station had the smallest elevation difference to each of our plots (Figure S4) and calculated Δ*T* accordingly for canopy and soil layers from corresponding plots, matching the respective monitoring time period.

### 2.7 Statistical analyses

We analyzed the relationship between the regional macroclimate, plot‐scale topographical, hydrological, and vegetation predictor variables and daily (1) microclimate temperatures within vegetation layers, and (2) Δ*T*s between vegetation layers. We focused on vegetation variables as predictors rather than responses, as previous studies identified consistent vegetation effects on microclimate (Kemppinen et al., 2021; Myers‐Smith & Hik, 2013; van Zuijlen et al., 2020), while acknowledging that microclimate also influences the establishment of vegetation (e.g., Niittynen et al., 2020). Analyses consisted of three sets of models, exploring (1) effects of proximal environment on microclimate (“microclimate models”), (2) consistency of growing season relationships of vegetation and microclimate across larger plots (“plot size models”), and (3) possibilities to extrapolate microclimatic conditions based on remotely sensed vegetation data (“extrapolation models”).

First, for microclimate models, we only included vegetation predictors for the small (0.79 m^2^) circles of each sample plot, as we expected them to best capture the hypothesized influence of vegetation on temperatures at the plot centers. We created two sets of explanatory variables, including either soil moisture (growing season) or snow cover duration (winter period) (Table S4), and tested for multicollinearity in our explanatory variables using Pearson correlation and Variance Inflation Factors (VIFs). We then used stepwise elimination of variables until all VIFs were <3 (Zuur et al., 2010). This excluded vegetation cover and height measured directly at the logger, as well as cover of litter and bare ground, as these were highly correlated with shrub cover or average vegetation height (Table S5). We also excluded slope inclination from winter models, as it was highly correlated with SRI for that period (*r* = −.83). We then scaled the variables prior to analysis by mean and standard deviation to make effect sizes comparable. We only retained plots with complete records for all explanatory variables, resulting in a sample size of *n* = 88 for growing season models and of *n* = 83 for winter period models, due to incomplete soil moisture and snow cover data, respectively (see above).

We analyzed relationships between predictor variables and local temperatures for separate layers as well as Δ*T*s between layers, incorporating all predictor variables into full models. To account for general dependency of temperatures on regional macroclimate, we included daily free‐air temperature from the lowest weather station in the study area as a reference free‐air temperature predictor. We used this record rather than the closest weather station for each plot (as for calculating temperature differences) to preserve general relationships of microclimate with elevation. All other predictors were included at seasonal constants.

Second, for plot size models, we followed the same steps as for microclimate models, however, using vegetation data derived from medium and large circles (Figure 1b) and only modeling growing season soil versus canopy Δ*T*. For the large circles, we excluded two additional plots with missing values for vegetation height (*n* = 86).

Third, for extrapolation models, we used plot‐level kNDVI, which was available for all plots (*n* = 90), as a single fixed‐effect predictor for soil versus canopy Δ*T*.

To control for pseudoreplication from daily temperature data, we included random intercepts for plot identity in our models, while all other predictors were included as fixed effects. We fitted linear mixed models in a Bayesian framework, applying the MCMCglmm package (Hadfield, 2010). We used weakly informative priors for all model terms: default normal priors for the fixed effects, an inverse Wishart prior for the residuals, and a parameter expanded prior for the random effect. All models were run for 100,000 Markov Chain–Monte Carlo iterations (burn‐in = 70,000, thinning rate = 10). We assessed model convergence by examining trace plots and autocorrelation values, and calculated model fit as marginal *R* ^2^ (*R* ^2^_marg_) and conditional *R* ^2^ (*R* ^2^_cond_) following equations 26 and 30 in Nakagawa and Schielzeth (2013), respectively. We consider an effect as “significant” if the predicted 95% credible interval does not overlap with zero.

All data management and analyses were performed in R v4.1.1 (R Core Team, 2021).

## 3 RESULTS

**FIGURE 3:**
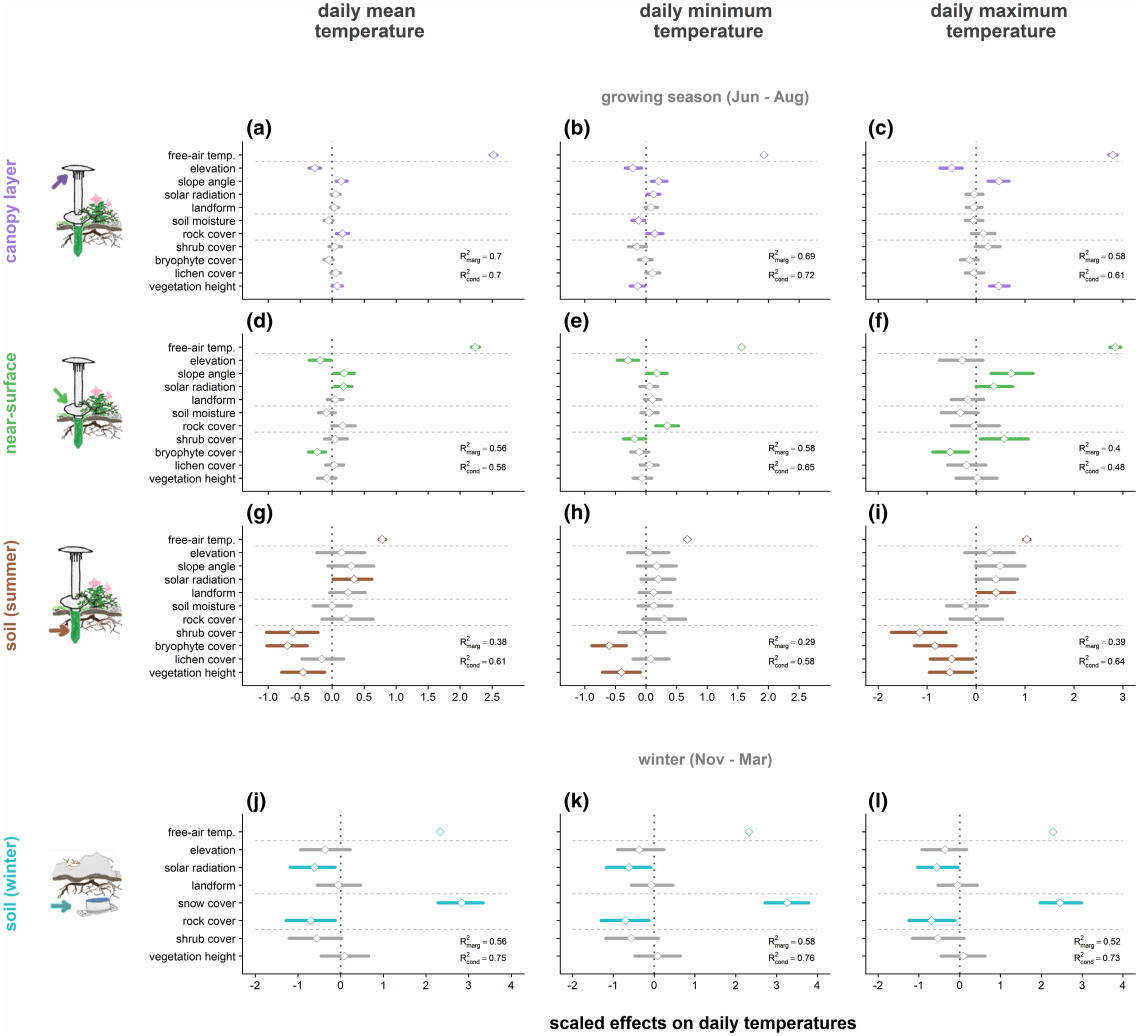
Tundra vegetation and snow cover duration were strong predictors of soil temperatures in particular, during the growing season and winter. Temperatures represent seasonal averages of daily mean, minimum, and maximum temperatures for (a–c) free‐air, (d–f) near‐surface, and (g–i) soil layers during the growing season, as well as for (j–l) soil during winter (November 2019 through March 2020). Points and horizontal segments represent means and 95% credible intervals for scaled effect sizes of plot‐scale topography and vegetation variables across small sample plots (radius 0.5 m). See Table S6 for predicted effect sizes for all models and variables.

**FIGURE 4:**
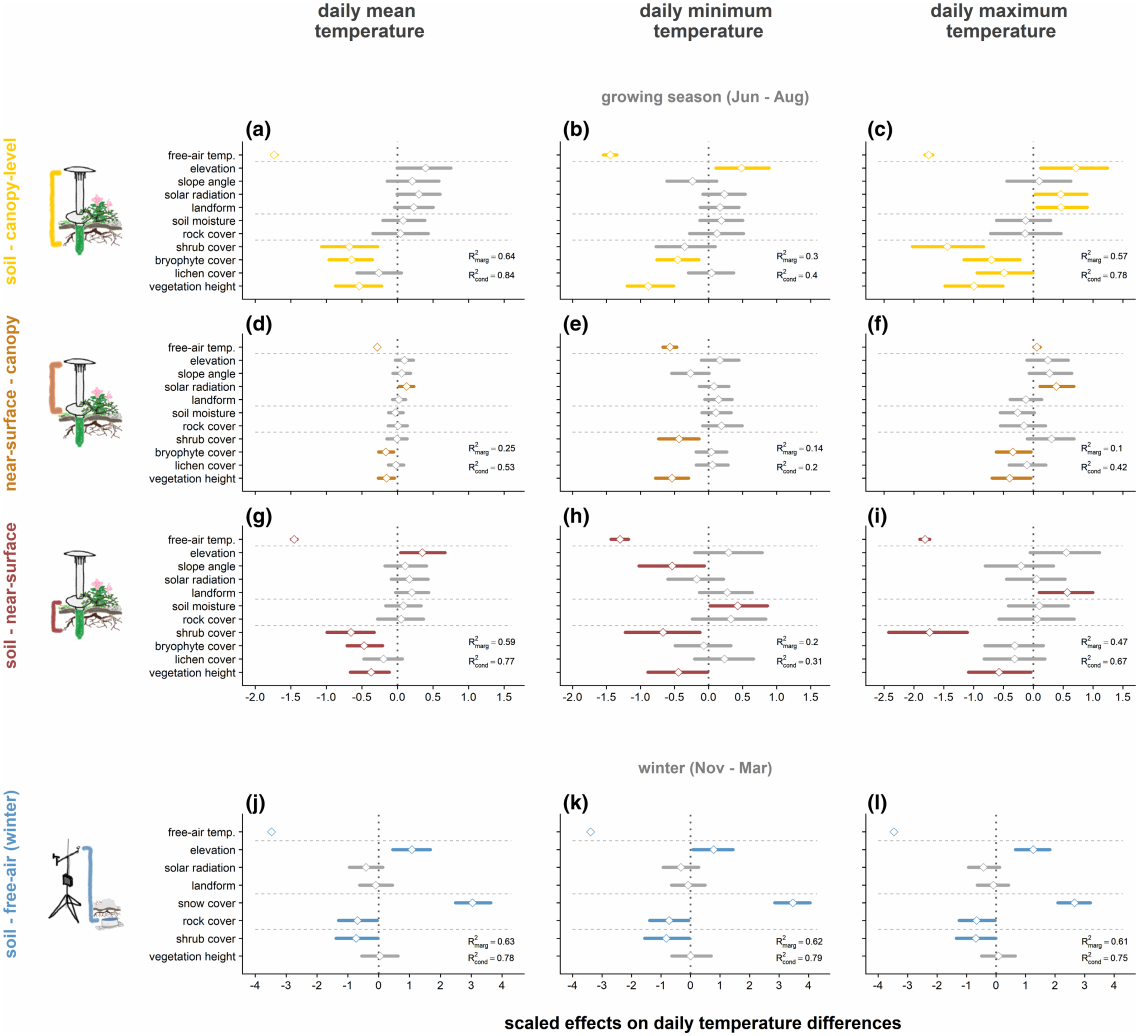
Vegetation predicted differences of soil temperatures relative to above‐ground temperatures better than local topography during the growing season, while snow cover duration was the strongest predictor during winter. Temperature differences were calculated between daily mean, minimum, and maximum temperatures for (a–c) canopy‐level versus soil, (d–f) canopy‐level versus near‐surface, and (g–i) near‐surface versus soil layers during the growing season of 2019, and for (j–l) free‐air versus soil layers during winter (November 2019 through March 2020). Points and horizontal segments represent means and 95% credible intervals for scaled effect sizes of plot‐scale topography and vegetation variables across small sample plots (radius 0.5 m). See Table S6 for predicted effect sizes for all models and variables, and Figure S5 for models of canopy versus free‐air and soil versus free‐air Δ*T*.

**FIGURE 5:**
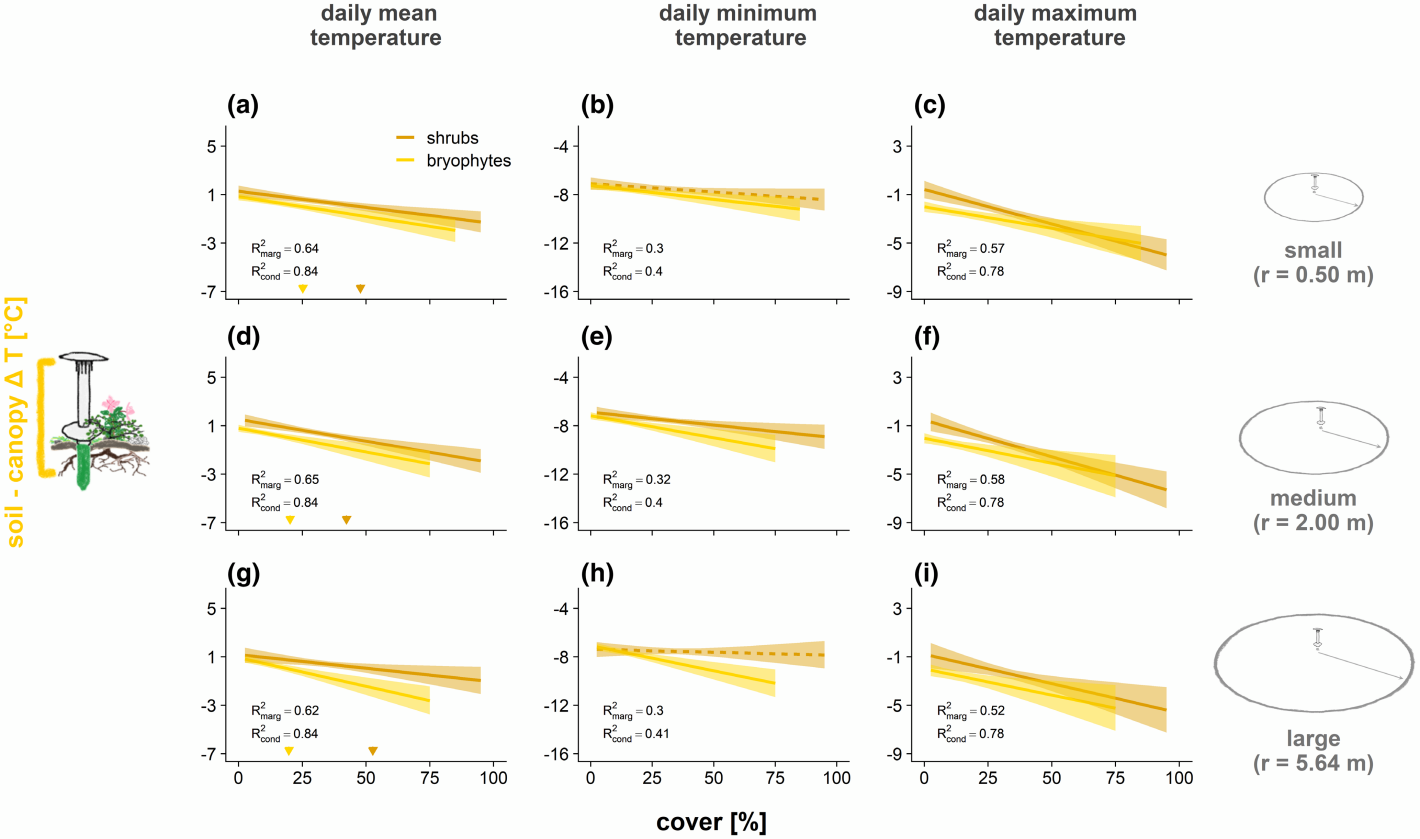
Growing season temperature differences between canopy level and soil shift from warmer to cooler soils with higher shrub and bryophyte cover. The relationships were consistent across plot sizes, as vegetation cover measures were highly correlated across plot sizes (Table S7). Predicted relationships of bryophyte and shrub cover on canopy versus soil differences in mean as well as average daily minimum and maximum temperatures over the 2019 growing season, compared across different plot sizes of (a–c) 0.5 m, (d–f) 2 m, and (g–i) 5.64 m radius. Dashed lines indicate nonsignificant relationships. Arrows indicate predicted cover values at which Δ*T*'s shift from warmer to cooler soils. Vertical axis ranges are aligned to make slopes comparable. Data points were removed to improve readability (presented in Figure S6). See also Table S7 for predicted zero‐difference thresholds for mean temperatures, and Figure S7 for corresponding predictions from vegetation height. Note that loggers and circle sizes on the right are not to scale.

Tundra microclimate was predominantly related to vegetation. Higher cover and taller vegetation significantly predicted colder soil temperature (*T*) during the growing season, but these relationships were weaker for near‐surface and canopy‐level *T* (Figure 3). This predicted cooling effect was also expressed in the daily temperature differences between vegetation layers (Δ*T*; Figure 4). Growing season Δ*T*s showed consistent and significant relationships with vegetation predictors across larger plot radii, shifting from relatively warmer soils at low cover to cooler soils underneath closed vegetation (Figure 5). During winter, warmer soils were associated with an extended snow cover duration (Figures 3 and 4). In contrast to the vegetation predictors and snow, other abiotic variables had weaker predictive power for *T* and Δ*T* (Figures 3 and 4). However, we point out that our analyses are based on correlations and thus do not test for causal relationships.

Growing season temperature extremes were generally buffered below‐ground, as daily temperature variation was smaller in soils relative to above‐ground vegetation layers (Figure 2). Accordingly, the association of daily mean (*T*_mean_), minimum (*T*_min_), and maximum temperatures (*T*_max_) with corresponding moments of regional free‐air *T* weakened from the canopy layer and near surface toward the soil level (Table 1, Figure 3). In addition, we recorded overall higher *T*_min_ and lower *T*_max_ in soils than in above‐ground vegetation layers (Figure S8).

**TABLE 1:** Mean posterior effect size estimates for relationships of free‐air temperature, topography, moisture, and vegetation variables with absolute daily mean, minimum, and maximum temperatures at different heights above and in Arctic tundra soil. Values were obtained from Bayesian linear mixed modeling and have been transformed to the respective units given for each variable. Blank cells indicate nonsignificant effects (i.e., 95% credible intervals overlapping with zero). Gray cells show variables not included as predictors for respective temperature responses

Variable	Unit	Canopy‐level temperature			Near‐surface temperature			Soil temperature (growing season)			Soil temperature (winter)		
		Mean	Min	Max	Mean	Min	Max	Mean	Min	Max	Mean	Min	Max
Free‐air temp.	°C/°C	0.86	0.75	0.89	0.76	0.61	0.91	0.27	0.27	0.33	0.37	0.36	0.37
Elevation	°C/100 m	−0.12	−0.09	−0.21	−0.08	−0.12							
Slope angle	°C/°	0.02	0.03	0.06	0.03	0.02	0.10						
Solar radiation	°C/% of seasonal max.		0.02		0.03		0.07	0.06			−0.09	−0.09	−0.09
Landform	—									0.47			
Soil moisture	°C/10% VWC		−0.09										
Snow cover duration	°C/10 days										0.75	0.86	0.65
Rock cover	°C/10% cover	0.09	0.08			0.20					−0.40	−0.40	−0.39
Shrub cover	°C/10% cover					−0.07	0.23	−0.24		−0.45			
Bryophyte cover	°C/10% cover				−0.12		−0.27	−0.35	−0.30	−0.42			
Lichen cover	°C/10% cover									−0.35			
Vegetation height	°C/cm taller vegetation	0.03	−0.05	0.17				−0.17	−0.15	−0.20			

Free‐air reference temperature was the strongest predictor of local temperatures and temperature differences in all growing season models. However, its importance and effect strength decreased from canopy layer to soil level (Figures 3 and 4; Tables 1 and 2). Model fit was therefore best for canopy level and lowest for soil *T* models (Figure 3), though accounting for sample plot identity explained particularly much variation in soil *T* (Figure 3g–i).

**TABLE 2:** Mean posterior effect size estimates for relationships of free‐air temperature, topography, moisture, and vegetation variables with differences in daily mean, minimum, and maximum temperatures between different heights above and in the ground in Arctic tundra. Values were obtained from Bayesian linear mixed modeling and have been transformed to the respective units given for each variable. Blank cells indicate nonsignificant effects (i.e., 95% credible intervals overlapping with zero). Gray cells show variables not included as predictors for respective temperature difference responses

Variable	Unit	Soil–canopy‐level Δ*T*			Near‐surface–canopy‐level Δ*T*			Soil–near‐surface Δ*T*			Soil–free‐air Δ*T* (winter)		
		Mean	Min	Max	Mean	Min	Max	Mean	Min	Max	Mean	Min	Max
Free‐air temp.	°C/°C	−0.59	−0.56	−0.56	−0.10	−0.22	0.02	−0.50	−0.51	−0.58	−0.56	−0.53	−0.57
Elevation	°C/100 m		0.20	0.30				0.15			0.46	0.34	0.54
Slope angle	°C/°								−0.07				
Solar radiation	°C/% of seasonal max.			0.09	0.02		0.07						
Landform	—			0.53						0.65			
Soil moisture	°C/10% VWC								0.33				
Snow cover duration	°C/10 days										0.81	0.92	0.70
Rock cover	°C/10% cover										−0.39	−0.41	−0.37
Shrub cover	°C/10% cover	−0.27		−0.57		−0.17		−0.26	−0.26	−0.68	−0.29	−0.33	−0.27
Bryophyte cover	°C/10% cover	−0.33	−0.23	−0.35	−0.08		−0.17	−0.24					
Lichen cover	°C/10% cover			−0.34									
Vegetation height	°C/cm taller vegetation	−0.20	−0.33	−0.37	−0.06	−0.20	−0.15	−0.14	−0.16	−0.21			

### 3.1 Vegetation predictors: Growing season temperature within different vegetation layers

After free‐air reference temperatures, vegetation variables were the strongest predictors for growing season T in the soil, but not in near‐surface and canopy layers (Figure 3, Table 1). Specifically, higher bryophyte cover and taller vegetation were associated with colder soils across temperature variables, while higher shrub cover was the strongest vegetation predictor of lower soil *T*_mean_ and *T*_max_ (Figure 3g–i). Remarkably, vegetation–soil temperature relationships were consistently negative, including for *T*_min_, indicating that vegetation contributed to buffering of *T*_mean_ and *T*_max_, but not *T*_min_ (Figure 3, Table 1). In contrast, vegetation–temperature relationships were considerably weaker or absent in above‐ground layers (Figure 3a–f, Table 1). Notably, and in disagreement with our hypothesis, higher shrub cover predicted higher *T*_max_ at near‐surface level (Figure 3f).

### 3.2 Vegetation predictors: Growing season temperature differences between vegetation layers

The stronger relationships between vegetation predictors and soil temperatures translated into stronger associations with temperature differences and higher model fit when comparing soil to above‐ground temperatures (Figure 4, Table 2). Higher cover of shrubs and bryophytes as well as taller vegetation predicted negative below‐ versus above‐ground Δ*T* as a result of lower soil compared to near‐surface and canopy‐level *T* (Figure 4a–c,g–i; Table 2), while higher lichen cover was only associated with lower soil versus canopy‐layer Δ*T*_max_ (Figure 4c). Importantly, soils were often warmer than canopy layers underneath low bryophyte and lichen cover, but colder underneath more closed canopies (Figure 5a–c), with the cover threshold for the shift from warmer to cooler soils being consistently higher for shrubs than bryophytes (51% vs. 29%; Table S7). Similarly, yet overall more weakly, taller vegetation predicted lower near‐surface versus canopy‐level Δ*T*, as did higher bryophyte cover for Δ*T*_mean_ and Δ*T*_max_, and higher shrub cover for Δ*T*_min_ (Figure 4d–f; Table 2).

**FIGURE 6:**
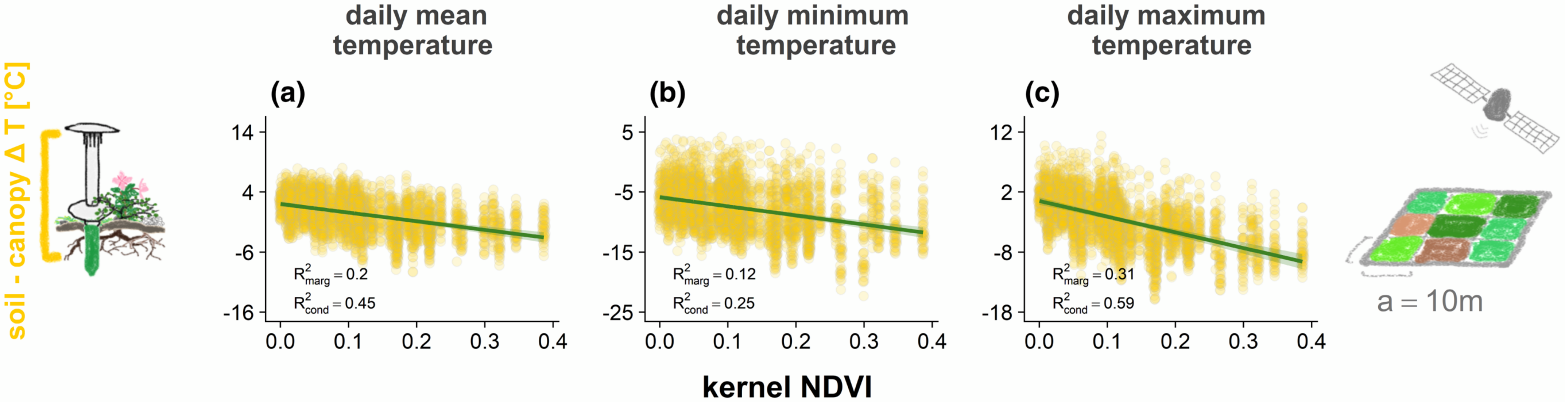
Remotely sensed vegetation productivity reliably predicted below‐ versus above‐ground Δ*T*, but explained too little variation for reliable extrapolation. Predicted linear relationships of kernel NDVI (Camps‐Valls et al., 2021) with canopy versus soil differences in daily (a) mean, (b) minimum, and (c) maximum temperatures over the 2019 growing season. We extracted kNDVI for large plots (100 m^2^; Figure 1b) from 10‐m resolution Sentinel‐2 imagery from July 29, 2019. Vertical axis ranges are aligned to make slopes comparable.

Contrasting our third hypothesis, relationships of shrub and bryophyte cover with soil versus canopy‐level Δ*T* did not weaken across larger sample plots (Figure 5) and cover thresholds for warmer versus cooler soils were overall consistent (Table S7), as values for both shrub and bryophyte cover were highly correlated across plot sizes (Pearson's *r* < .78; Table S8). This was also the case for vegetation height, though here, predicted effects slightly decreased in strength with larger plot size, and uncertainty was larger due to a skewed distribution of measured values (Figure S7). In the large plots, values of kNDVI were relatively highly correlated with shrub cover (Pearson's *r* = .74) and vegetation height (*r* = .60), but less so with bryophyte cover (*r* = .18). Consequently, while predicted relationships of soil versus canopy‐level Δ*T* with kNDVI confirmed the negative trends and were well constrained (Figure 6), the amount of variation in Δ*T* explained by kNDVI was too low (*R* ^2^_marg_ ≤ .31) to carry out meaningful extrapolations of microclimatic differences onto the landscape scale.

### 3.3 Abiotic predictors for growing season microclimate

Topography, rock cover, and soil moisture predictors were more important predictors for growing season temperatures at canopy and near‐surface level than in soils (Figure 3, Table 1). For example, canopy‐level *T* was lower at higher elevation and on gentle slopes, but neither elevation nor slope angle significantly predicted variation in soil *T* (Figure 3). Soil versus canopy‐level Δ*T*_min_ and Δ*T*_max_ were therefore higher at higher elevation (Table 2), but more variable in relation to slope angle, solar radiation, landform, soil moisture, and rock cover. The same was the case for comparisons among other vegetation layers (Figure 4). The strength of relationships between abiotic predictors and growing season microclimate was generally low, and similar or lower than for vegetation variables (Figures 3 and 4; Tables 1 and 2). Consequently, no abiotic variable emerged as a consistent predictor of growing season microclimate across temperature variables, neither for *T* nor Δ*T*.

### 3.4 Predictions of winter temperature and temperature difference

Across all sample plots, daily soil temperature amplitude was considerably lower between November and March than during the growing season (Figure 2). Both free‐air and soil temperature were mostly negative during the winter, while soils were mostly warmer than free‐air conditions (Figures S8 and S9).

We recorded higher winter soil temperatures with warmer macroclimate (Table 1, Figure 3j–l), but lower soil versus free‐air Δ*T* (Table 2, Figure 4j–l) with warmer regional free‐air temperature. Yet, the strongest predictor for higher winter soil temperatures was a longer duration of snow cover (Table 1, Figure 3j–l), which was mirrored in a strong positive relationship of snow cover duration with soil versus free‐air Δ*T* (Table 2, Figure 4j–l). In contrast, higher cover of rocks predicted lower soil T and lower soil versus free‐air Δ*T* in all temperature variables (Figures 3 and 4j–l). Higher solar radiation was associated with slightly lower winter soil *T* (Figure 3j–l), while higher elevation had a positive and higher shrub cover a negative relationship with soil versus free‐air Δ*T* (Figure 4j–l). Neither elevation or shrub cover was significantly related to winter soil *T*, and slope angle, landform, and vegetation height did not predict variation in either winter soil *T* or Δ*T*.

## 4 DISCUSSION

Our results highlight the important role of local tundra vegetation as well as snow and rock cover in mediating the temperature regimes experienced by organisms close to the ground and in the topsoil. During the growing season, dense vegetation was associated with significantly lower soil temperatures compared to canopy‐level temperatures, while both low vegetation cover during summer and an extended snow cover during the winter months (November–March) were connected to warmer soils. In comparison to vegetation variables, abiotic predictors explained considerably less variation in growing season temperature regimes. Contrary to our expectations, we found no effect of spatial resolution (0.8–100 m^2^ plot size) on the relationships between shrub and bryophyte cover and soil‐canopy temperature differences. Although this suggests a pathway for extrapolating tundra microclimate based on remotely sensed vegetation data, more research will be needed to explore additional data sources and improve the quality of predictions. Our findings suggest that future vegetation changes might have important consequences for soil temperature in tundra environments, in turn influencing the composition and functioning of Arctic plant and soil microbial communities.

Higher cover and taller vegetation predicted lower soil temperatures during the growing season, with shrubs and bryophytes showing the strongest negative effects (Figure 3g–i), likely through alternate effects on radiative, convective, or latent heat exchange. Previous studies have also demonstrated that denser shrub canopies induce summer cooling of Arctic tundra soils (Aguirre et al., 2021; Blok et al., 2010; Kemppinen et al., 2021; Myers‐Smith & Hik, 2013), as more closed and taller canopies shade the soil and prevent input of radiative or convective energy into the below ground system, thereby lowering soil temperatures (Myers‐Smith et al., 2011). Meanwhile, bryophyte mats cool soils through increased latent heat transfer from the soil through their high water‐holding capacity (Beringer et al., 2001; Gornall et al., 2007). Although not studied here, the thickness of bryophyte mats might be an important determinant of local soil temperature as thickness represents the main control on water retention (Gornall et al., 2007; Soudzilovskaia et al., 2013). In addition to shrubs and bryophytes, lichens can provide another barrier for energy transfer into the soil due to their high reflectivity (Bernier et al., 2011) and intermediate water‐holding capacity (approx. 50%–75% of that of bryophytes; van Zuijlen et al., 2020). These barriers may especially contribute to the negative association of lichen cover with maximum soil temperatures as found in this study (Figure 3i) and by Mallen‐Cooper et al. (2021), while the stable relationship with minimum soil temperatures might point to a higher importance of reflectivity than water retention. Litter, which was highly correlated with shrub cover (Table S5), or other surface organic material may also have contributed to determining soil microclimate (Frost et al., 2018). In comparison, variables representing topography or moisture were considerably weaker predictors of soil temperatures (Figure 3g–i), suggesting that tundra vegetation plays a more important role in determining below‐ground microclimatic conditions. Notably, our findings suggest that the impacts of different vegetation types on soil temperatures occurred largely independently from each other within the landscape, as none of the vegetation predictors were highly correlated (Table S5). Different functional types of tundra vegetation within a landscape may thus contribute to the cooling of soil microclimates through their complementary effects on different parts of the energy budget (Heijmans et al., 2022).

Our findings show that the effects of surface cover on energy transfer are also important during the winter period (November–March). Extended snow cover duration predicted warmer soil temperatures, and higher rock and lichen presence were associated with colder soils (Figure 3j–l). Snow provides a thermal barrier between the soil and cold winter air through its air‐filled structure (e.g., Zhang, 2005). However, if the snow is sufficiently shallow, rocks can provide a conductive pathway for energy transfer from soil to air, resulting in cooling of soils (Harris & Pedersen, 1998). More intense solar radiation might contribute to maintaining a thinner snow cover, and hence to lower soil temperatures (Figure 3j–l). Surprisingly and unlike previous studies, we did not observe a positive correlation between our estimates of snow cover duration and shrub cover (see e.g., Aguirre et al., 2021; Grünberg et al., 2020; Myers‐Smith & Hik, 2013; Sturm et al., 2001) or vegetation height (Paradis et al., 2016; Table S5), instead shrub cover was rather associated with lower winter soil temperatures (Figure 3j–l). One could expect a closer relationship between shrub presence and canopy height with snow depth rather than duration of snow cover, and snow depth might be a more important determinant for winter soil temperatures (Aalto et al., 2018; Way & Lapalme, 2021; Way & Lewkowicz, 2018). We recommend that future studies monitor snow depth and density throughout winter and shoulder seasons to obtain a comprehensive understanding of its net insulating effect and identify any implications for below‐ground biological activity. The development of improved remote sensing products of snow depth (e.g., Broxton et al., 2019) could facilitate such undertakings where in‐field measurements are logistically challenging.

For both growing season and winter, the predictive strength of vegetation parameters was weaker for above‐ground temperatures than for soil temperatures. Accordingly, vegetation parameters were also significant predictors for Δ*T*s between these layers in our analysis (Figure 4). Our results support previous findings from monitoring of temperature profiles between contrasting vegetation types in North American tall‐shrub tundra (Kade et al., 2006; Klene et al., 2001) and Antarctica (Cannone & Guglielmin, 2009), as well as across topographically heterogeneous landscapes (Aalto et al., 2018). These studies also demonstrated the ability of vegetation and snow to affect soil temperatures specifically, thereby creating thermal differences across the vegetation column. Our study confirms these findings for the continuous variation of environmental conditions across the tundra landscape at our field site on Disko Island. Furthermore, our findings show consistent patterns across multiple environmental gradients, highlighting the importance of local environments in regulating microclimates and illustrating the limited relevance of free‐air temperatures for organisms living close to or in the ground. Thus, we should explicitly consider the influence of soil temperature patterns on organismal responses in research that aims to accurately forecast future changes in the rapidly warming Arctic tundra (Lembrechts et al., 2019).

Contrary to our expectations, we observed stable relationships of vegetation cover with Δ*T*s across plot sizes (Figure 5), indicating that vegetation structure and composition did not vary substantially across the ~10 m scale of our nested plots. Yet, while aligning well with shrub cover across the corresponding large sample plots, Sentinel‐2‐derived kNDVI did not reflect cover of all plant functional types equally well. Therefore, kNDVI did not capture the contrasting aspects of the energy budget that determined soil microclimate, which limited its predictive power for soil versus above‐ground Δ*T* (Figure 6). To improve the quality of predictions, we encourage exploration of additional remotely sensed predictors reflecting different plant functional types. Especially studies covering an even wider variety of vegetation structure than present at our study site (e.g., including larger areas of tall‐shrub tundra) and including other gridded variables (Raynolds et al., 2006) should improve spatial predictions of tundra microclimates at landscape and potentially larger extents. While further ground truthing through in situ microclimate measurements will be needed, such efforts might facilitate microclimate predictions especially for study areas with limited accessibility. They could thus add valuable knowledge to existing links between remote sensing data, local microclimate measurements, and fine‐scale vegetation structure (Zellweger et al., 2019).

Local alteration of the soil microclimate underneath shrub‐ or bryophyte‐dominated vegetation has considerable implications for tundra ecological processes and communities. Bryophytes can reduce seedling survival through creation of colder microclimates, thus impacting community composition (Lett et al., 2020; Soudzilovskaia et al., 2011; Vandvik et al., 2020) and potentially limiting expansion of non‐native species (Lembrechts et al., 2018). In addition, during the growing season, temperature‐dependent soil processes such as microbial decomposition or root respiration can be considerably slower underneath certain vegetation types (Ward et al., 2015), potentially also decreasing gas exchange rates and long‐term nutrient availability (Cahoon et al., 2012). Colder microclimates underneath dense vegetation may also contribute to restricting permafrost thaw during summer (Blok et al., 2010). However, elevated winter temperatures underneath snow cover may outweigh summer cooling in tundra vegetation (Way & Lapalme, 2021) and increase microbial and enzyme activity (Mikan et al., 2002; Wallenstein et al., 2009), nitrogen mineralization (Schimel et al., 2004), decomposition rates (Schimel et al., 2004), and active layer depth during winter (Heijmans et al., 2022; Lawrence & Swenson, 2011). In addition, these temperature‐mediated effects will likely add to more direct biotic influences of denser shrub vegetation on below‐ground biota in the Arctic (Myers‐Smith & Hik, 2013). These include increased soil microbial biomass as well as lower nitrogen availability (Aguirre et al., 2021) or shifts in microbial community composition (Parker et al., 2021), with potentially important implications for nutrient and carbon cycling. Quantifying the net effect of variation in vegetation and topography across seasons and scales will be crucial to determine future ecosystem responses and feedbacks in a changing Arctic.

As high‐latitude regions are experiencing rapid warming and prominent changes in vertical and horizontal vegetation structure (Bjorkman et al., 2018), ensuing alterations of microclimatic controls on species establishment, microbial processes, nutrient cycling, and permafrost could potentially be relevant for the tundra biome as a whole. The dominant vegetation type at our study site, short‐statured, shrub‐dominated tundra, covers around 29.5% of the terrestrial Arctic (Raynolds et al., 2019). Our findings suggest that expansion of this vegetation type could partly offset atmospheric warming for tundra soil environments during the growing season. In contrast, the transition of existing dwarf‐shrub to tall‐shrub tundra, facilitating snow accumulation, could eventually reverse the growing season soil cooling effect into net annual soil warming (Heijmans et al., 2022; Kropp et al., 2021; Paradis et al., 2016). Also, local disturbance from extreme climatic events and peaks of insect herbivory (Bjerke et al., 2017), degrading permafrost (Lara et al., 2018) or fire (Rocha et al., 2012), could reduce shrub and bryophyte cover and induce crucial warming of soil microclimates (Figure 5; Gornall et al., 2007; Myers‐Smith & Hik, 2013; Soudzilovskaia et al., 2013). In addition, a decrease or thinning of bryophyte mats could reduce water retention, facilitating establishment of vascular plants. Changes in both shrub cover and height, as well as changes in bryophyte cover or thickness, could therefore affect vegetation change through altering seedling survival, or soil microbial and nutrient dynamics (Cahoon et al., 2012; Vandvik et al., 2020). Furthermore, these vegetation dynamics will likely control tundra permafrost dynamics (Heijmans et al., 2022). However, considerable temporal and spatial heterogeneity of vegetation changes, as well as interacting factors such as snow, soil moisture or substrate quality, complicate precise forecasts of vegetation developments (see e.g., Elmendorf et al., 2012; Heijmans et al., 2022; le Roux et al., 2013). We therefore encourage large‐scale stratified random monitoring approaches including these factors to reduce such uncertainties and disentangle interactive effects of environmental factors on microclimate. This will enable a better understanding of fine‐scale variation in tundra vegetation across multiple gradients, as well as potential ecosystem responses in changing Arctic environments.

## 5 CONCLUSION

We monitored year‐round local temperatures at different heights across an Arctic tundra landscape, with plots placed stratified randomly to cover gradients of topography, soil moisture, and vegetation productivity. During both summer and winter, vegetation and snow cover variables were strong predictors of microclimate, particularly in the soil, likely through their complementary effects on soil energy fluxes. This highlights the necessity to account specifically for effects of vegetation dynamics on soil thermal conditions in studies of soil‐dwelling biota and soil ecological processes in the tundra. Depending on future vegetation dynamics, these relationships can have crucial consequences for plant community composition, soil biotic activity, and ecosystem processes. As vegetation parameters predicted above‐below ground temperature differences equally well when sampled at different resolutions, remotely sensed data could help to predict these consequences at larger spatial extents. However, identifying additional predictors will be necessary to obtain reliable extrapolations.

## AUTHOR CONTRIBUTIONS

Jonathan von Oppen, Jakob J. Assmann, Urs A. Treier, Bo Elberling, Jacob Nabe‐Nielsen, and Signe Normand designed the study. Jonathan von Oppen, Jakob J. Assmann, Anne D. Bjorkman, Urs A. Treier, and Signe Normand collected field data. Jonathan von Oppen analyzed the data with support from Jakob J. Assmann, Anne D. Bjorkman, and Signe Normand. Jonathan von Oppen wrote the manuscript draft with input from all authors. All authors contributed to manuscript revisions.

## CONFLICT OF INTEREST

The authors declare that they have no conflict of interest.

## Supporting information


Appendix S1

## Data Availability

The data that support the findings of this study are openly available in zenodo at https://doi.org/10.5281/zenodo.7060023.
