# Supplementary material for: Cross‐scale regulation of seasonal microclimate by vegetation and snow in the Arctic tundra
Source: Glob Chang Biol. 2022 Sep 24;28(24):7296–312. doi: 10.1111/gcb.16426 (PMC9826065; doi:10.1111/gcb.16426)
Supplement: Supplementary file 1 — Appendix S1 [file GCB-28-7296-s001.pdf]

Cross-scale regulation of seasonal microclimate by vegetation and snow in the Arctic tundra

von Oppen J. et al. 2022 *Global Change Biology*

Supplementary note

Stratified random placement of sample plots

For placement of scattered plots, we first computed one random candidate location for each of the six classes (three vegetation x two moisture classes; Table S1) within each elevation band on each mountainside. Depending on availability within a class, we additionally computed up to five alternative random locations for each of these plots, resulting in 60 candidate and ~300 alternative locations. Furthermore, we placed an additional 30 plots located in clustered “core-areas'' on the eastern mountain slope to include high environmental variation within small spatial scales. Candidates for the core areas were selected from the list of all candidate sampling locations for the scattered plots (incl. alternatives) by choosing locations for which all six sampling classes of the landscape stratification were present within a 60 m radius around the location. Thereafter, we removed these core area locations from the lists of original locations - while maintaining their random order - and selected the first core area candidate as the prime candidate and the rest as alternatives. We then applied the random stratified sampling again, this time to generate an additional five locations within the 60 m radius around each potential core area centre by sampling the remaining five classes not covered by the centre location itself.

In the field, we established the sample plots based on the candidate locations where possible, working through the list of alternatives where lack of access, human disturbance (e.g. a path) or placement of the logger was not possible (see below for more details). We used handheld GPS devices (GARMIN eTrex 30, Garmin Ltd., Schaffhausen, Switzerland) to find plot locations with the predefined coordinates. Once the GPS reported arrival at the location, we placed a metal peg to mark the centre of the plot. In cases where placement of the peg and/or both loggers was not possible (e.g. due to shallow soil, solid rock, or standing water), we repeated the attempt

50 cm north, east, south and west of the original location. If necessary, further attempts were taken at 100, 150, and 200 cm in all cardinal directions. If placement was still not possible, we skipped the location and repeated the procedure at the next alternative location. In two cases, this resulted in placement of sample plots on the eastern instead of the western mountain slope, but in the same elevation band.

Hindsight assessment of vegetation cover across intended productivity classes confirmed that this part of the stratification was successful (Figure S10a). In contrast, comparison of local growing-season mean soil moisture across NDWI classes revealed that our study design did not systematically capture differences in water availability (Figure S10b). However, this mismatch could partly be due to between-year moisture variation, as the growing season of 2018 saw much more precipitation than the rather dry summer of 2019.

Supplementary figures

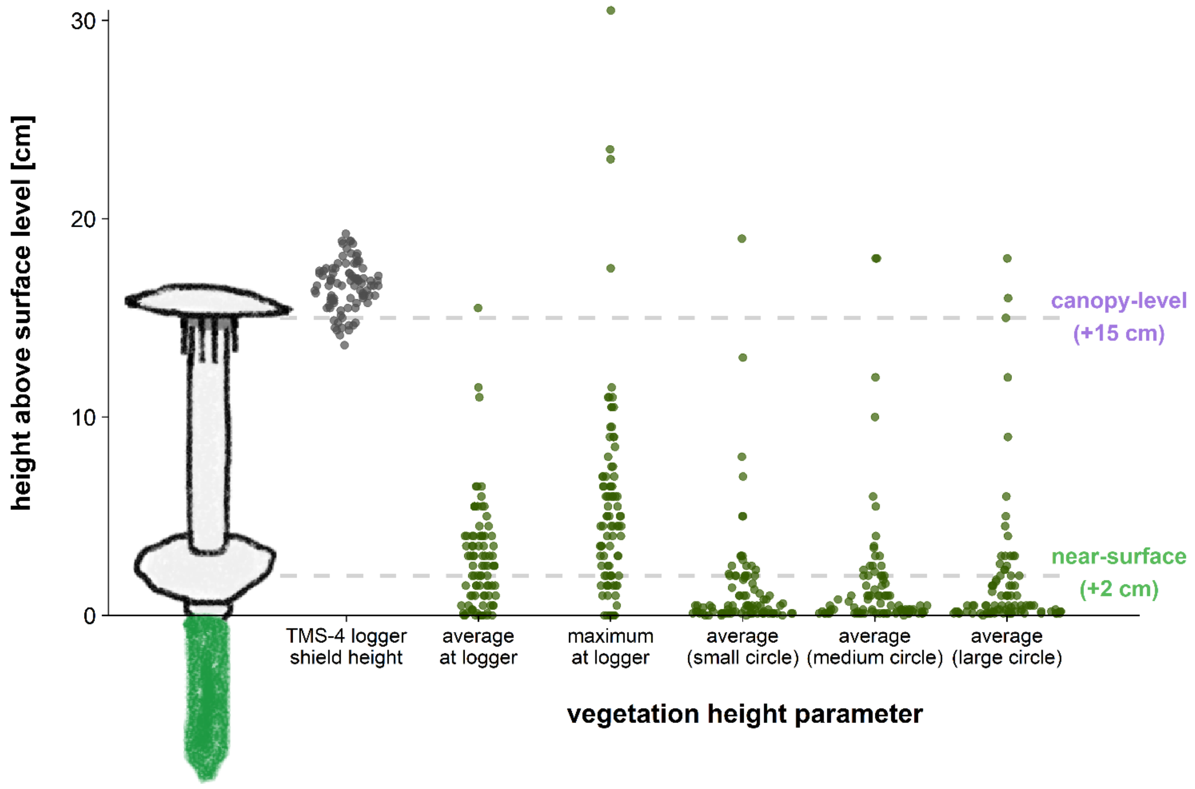

Figure S1:

The top (= air) sensor of the microclimate logger reached above the vegetation in most plots. Dots show the distributions of height measurements for the top shield of the TOMST TMS-4 logger and for vegetation at the logger as well as in each sampling circle (see Figure 1 in the main text). Dashed horizontal lines indicate position of the above-ground temperature sensors on the logger for reference.

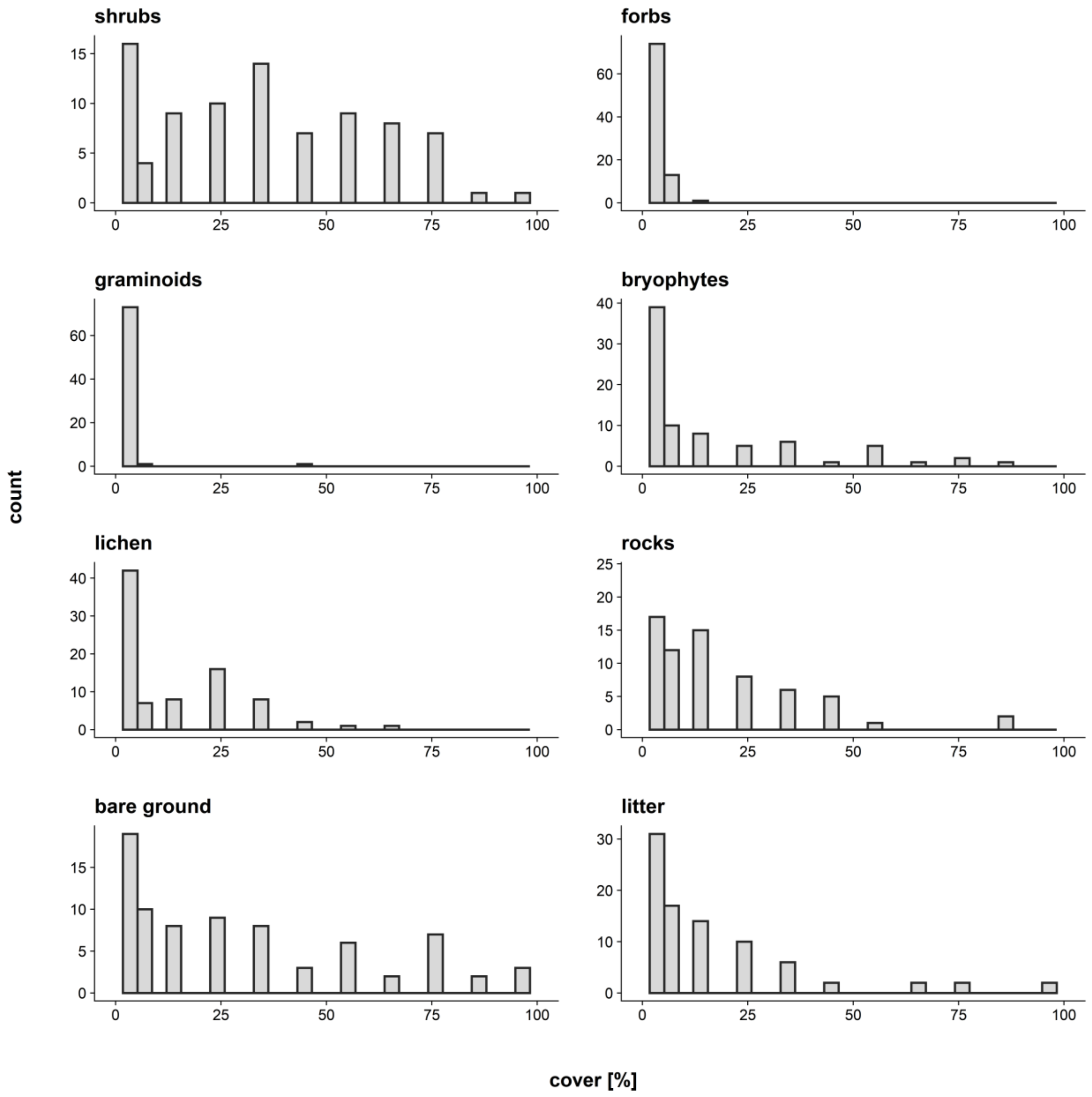

Figure S2:

Histograms of cover values for different categories in sample plots. Scarce forb and graminoid cover were excluded from analysis.

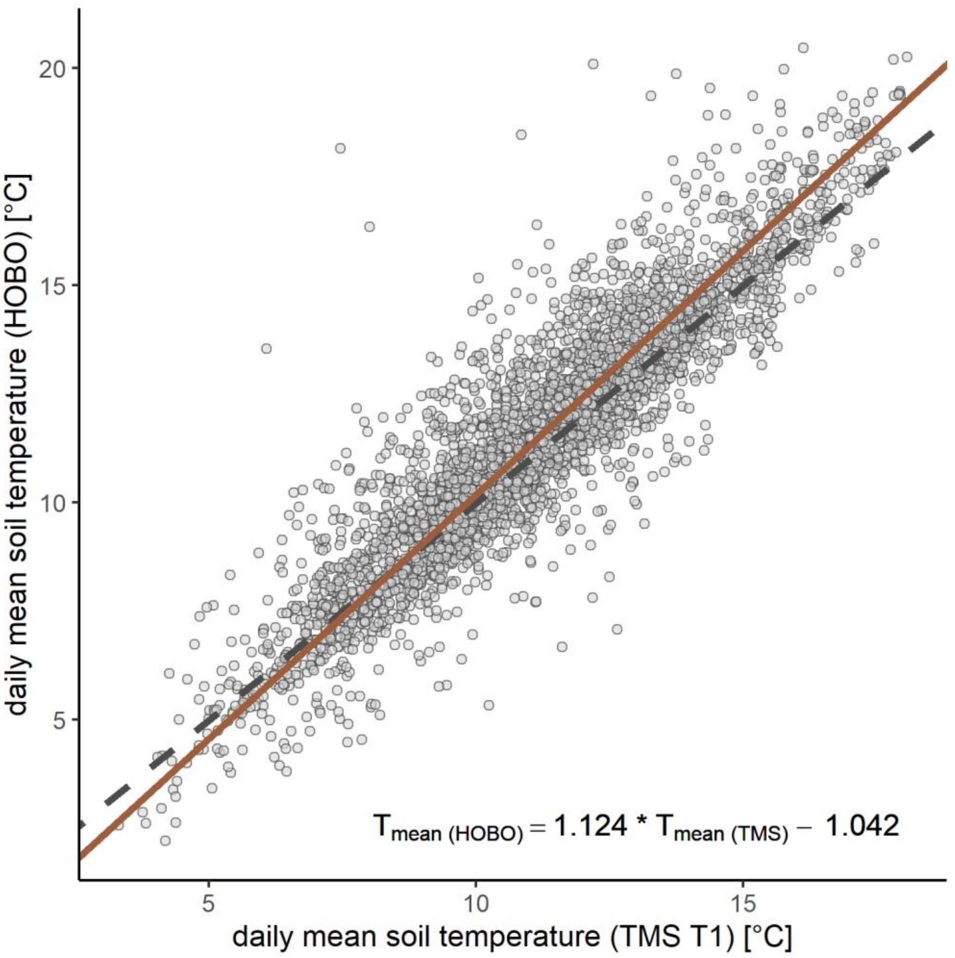

Figure S3:

Growing-season soil temperature measurements were well aligned between TMS and HOBO sensors. Points represent measurement pairs of daily mean temperature. The brown line shows the fitted linear relationship (coefficients shown in equation), and the grey dashed line presents the 1:1 relationship for comparison.

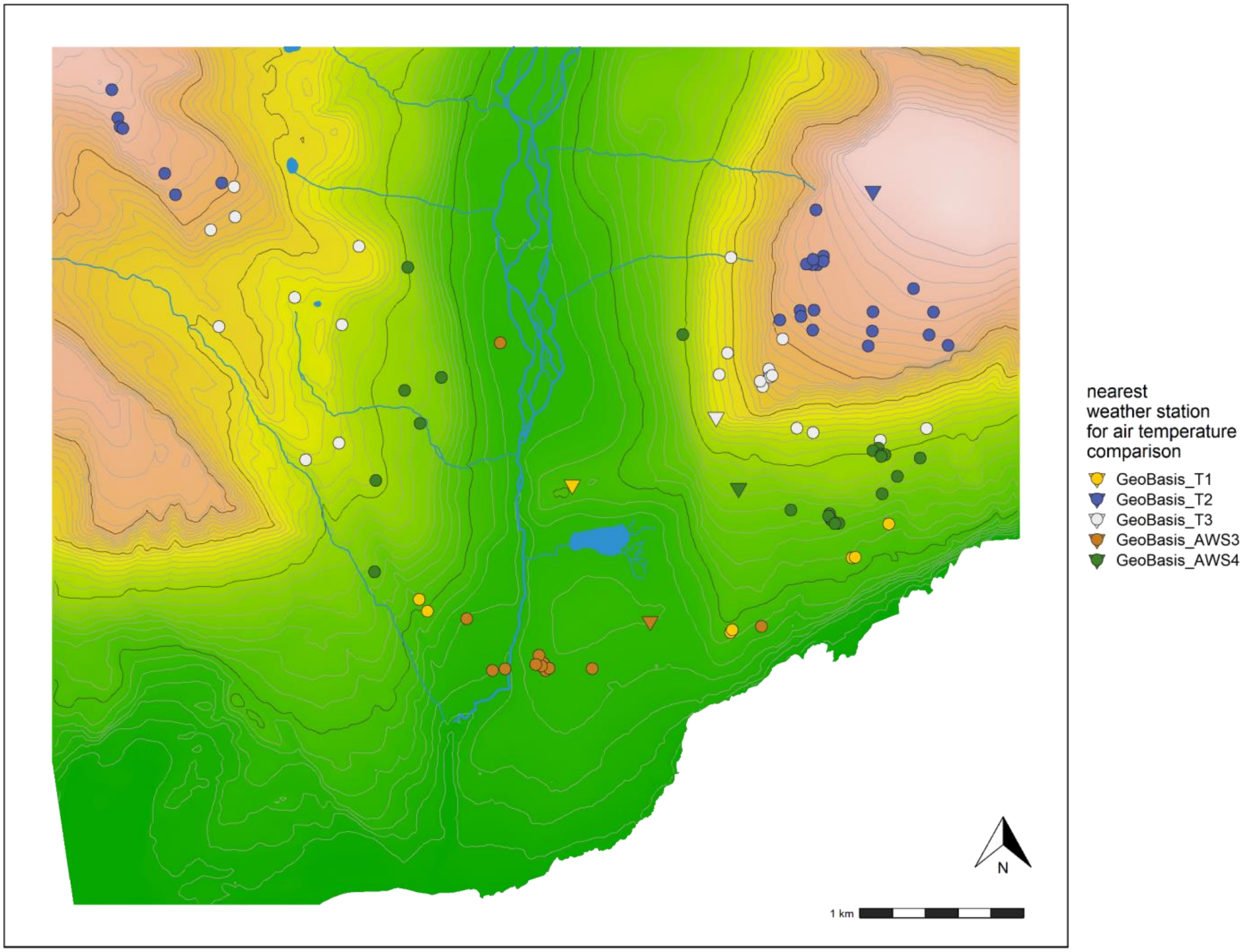

Figure S4:

Weather station with the smallest elevation difference to each sample plot, of which the free-air temperature time series was used to calculate temperature differences. See also Figure 1 in the main text for a description of plot placement.

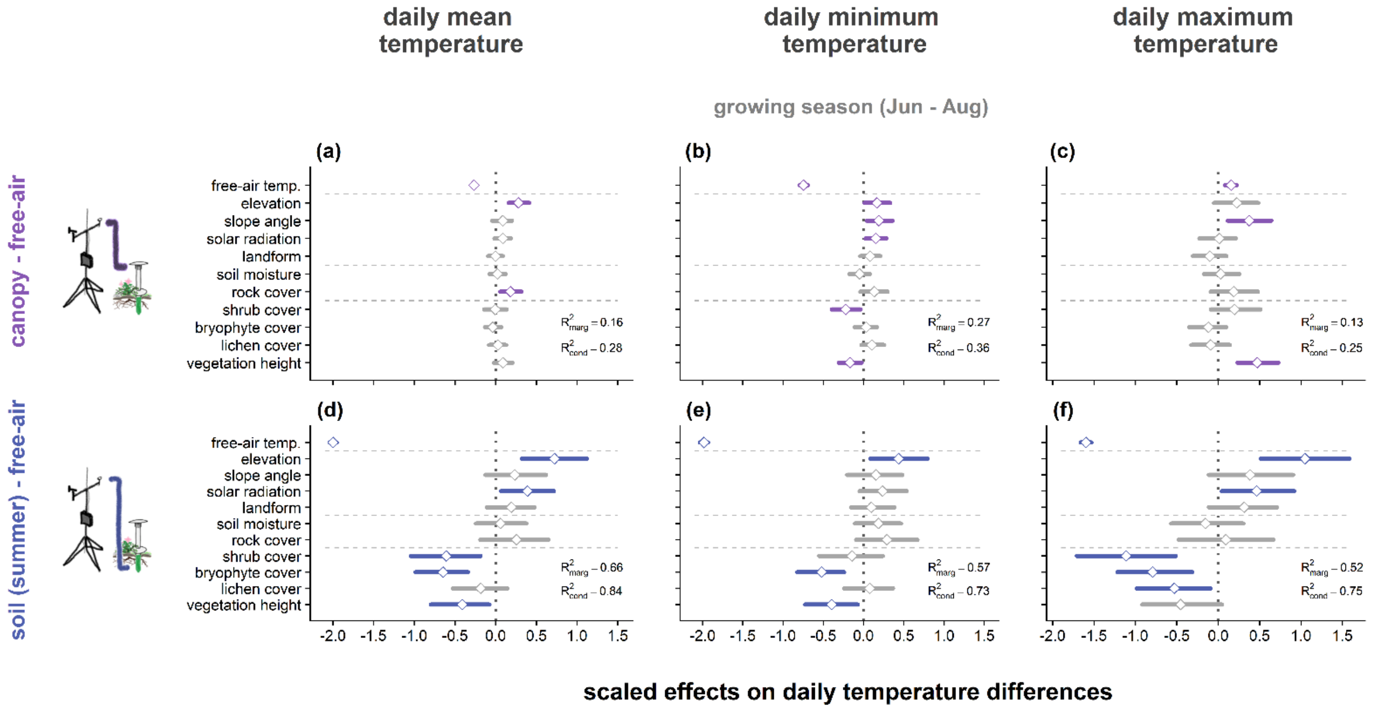

Figure S5:

Local factors weakly influenced differences between free-air weather stations and TMS canopy sensors **(a-c)**, resulting in relationships of soil vs. free-air ΔT with environmental variables largely consistent with those of soil vs. canopy-layer ΔT (**d-f**; compare Figure 4a-c in main text). Exceptions include significant positive relationships of soil vs. free-air ΔT_{mean} with elevation and solar radiation, as well as a non-significant relationship of soil vs. free-air ΔT_{max} with more exposed landforms and vegetation height. These differences might be partly attributable to elevational and topographical mismatches between plot and weather station locations (see Figure S4).

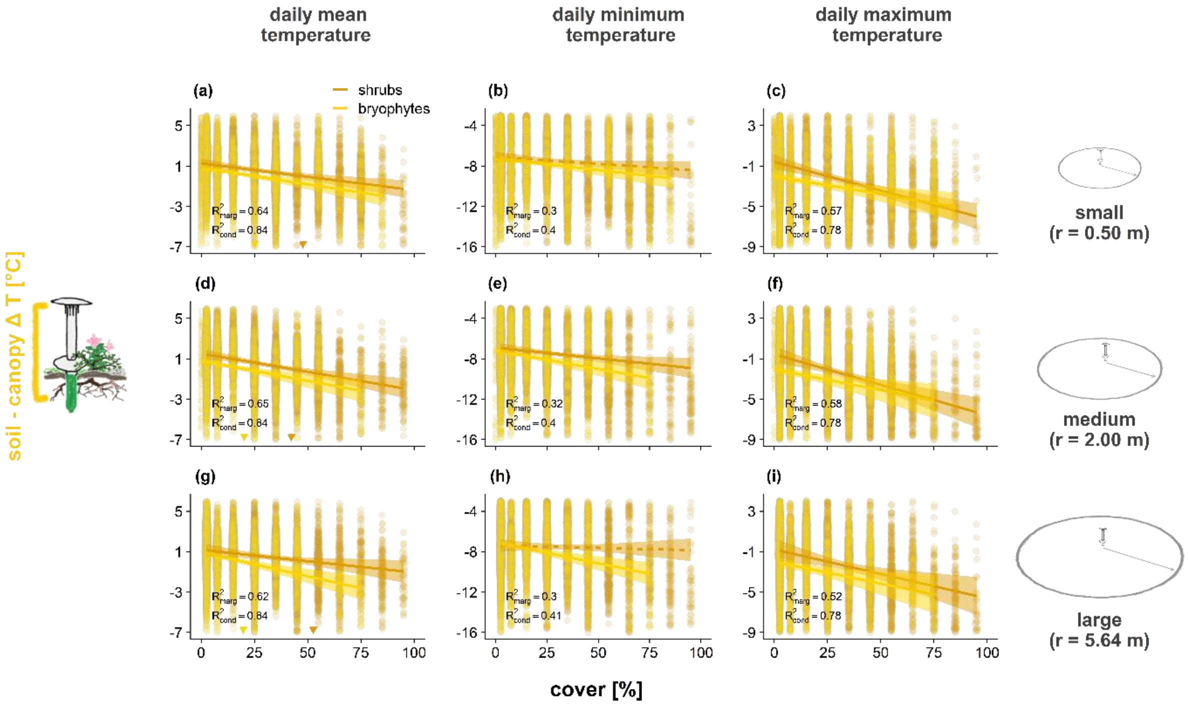

Figure S6:

Growing season temperature differences between canopy level and soil shift from warmer to cooler soils with higher shrub and bryophyte cover. The relationships were consistent across plot sizes, as vegetation cover measures were highly correlated across plot sizes (Table S7). This figure is identical to Figure 5 in the main text, but also shows the data points for daily temperature variables for each plot.

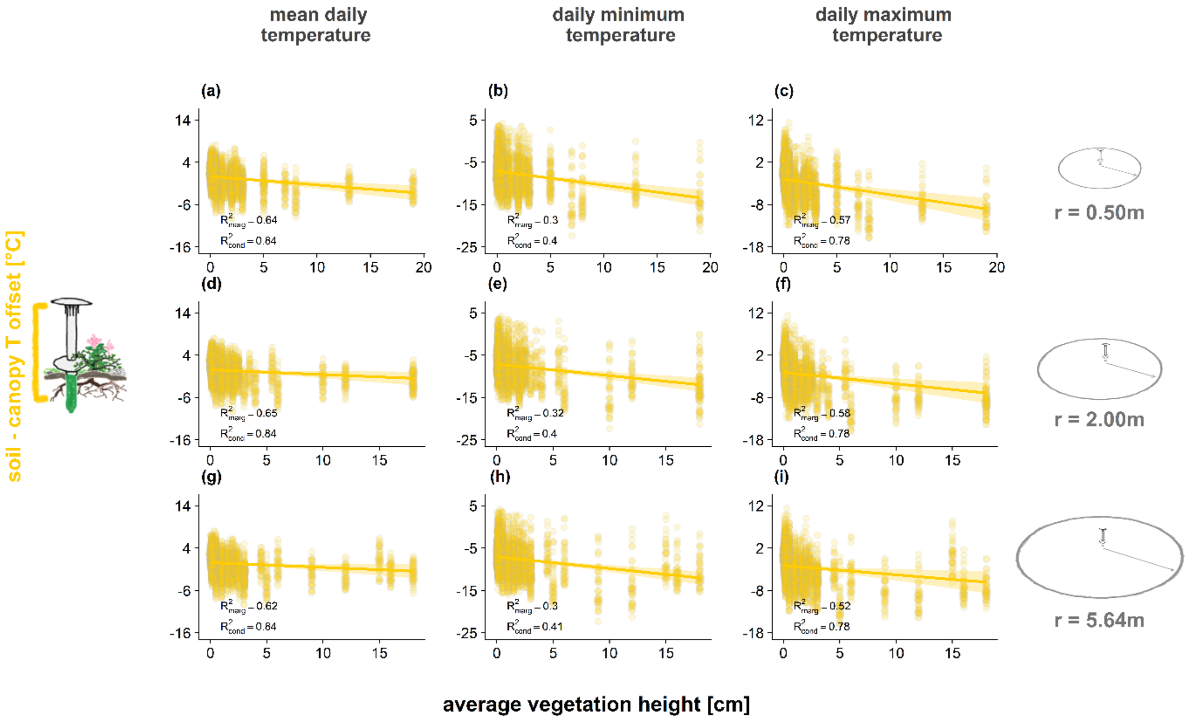

Figure S7:

Growing season temperature differences between canopy level and soil increase with taller vegetation. As height values were highly correlated (Table S7), this relationship was consistent across plot sizes. The panels show predicted relationships (solid green lines with light-green 95% credible intervals) between the average vegetation height within a plot and the observed difference between soil and canopy-level temperatures for the daily mean, minimum and maximum temperatures over the 2019 growing season. We measured the average vegetation height for three different sizes of circles around the centre of the plots; these circles had radii of **(a-c)** 0.5 m, **(d-f)** 2 m and **(g-i)** 5.64 m. Note that loggers and circle sizes on the right are not to scale.

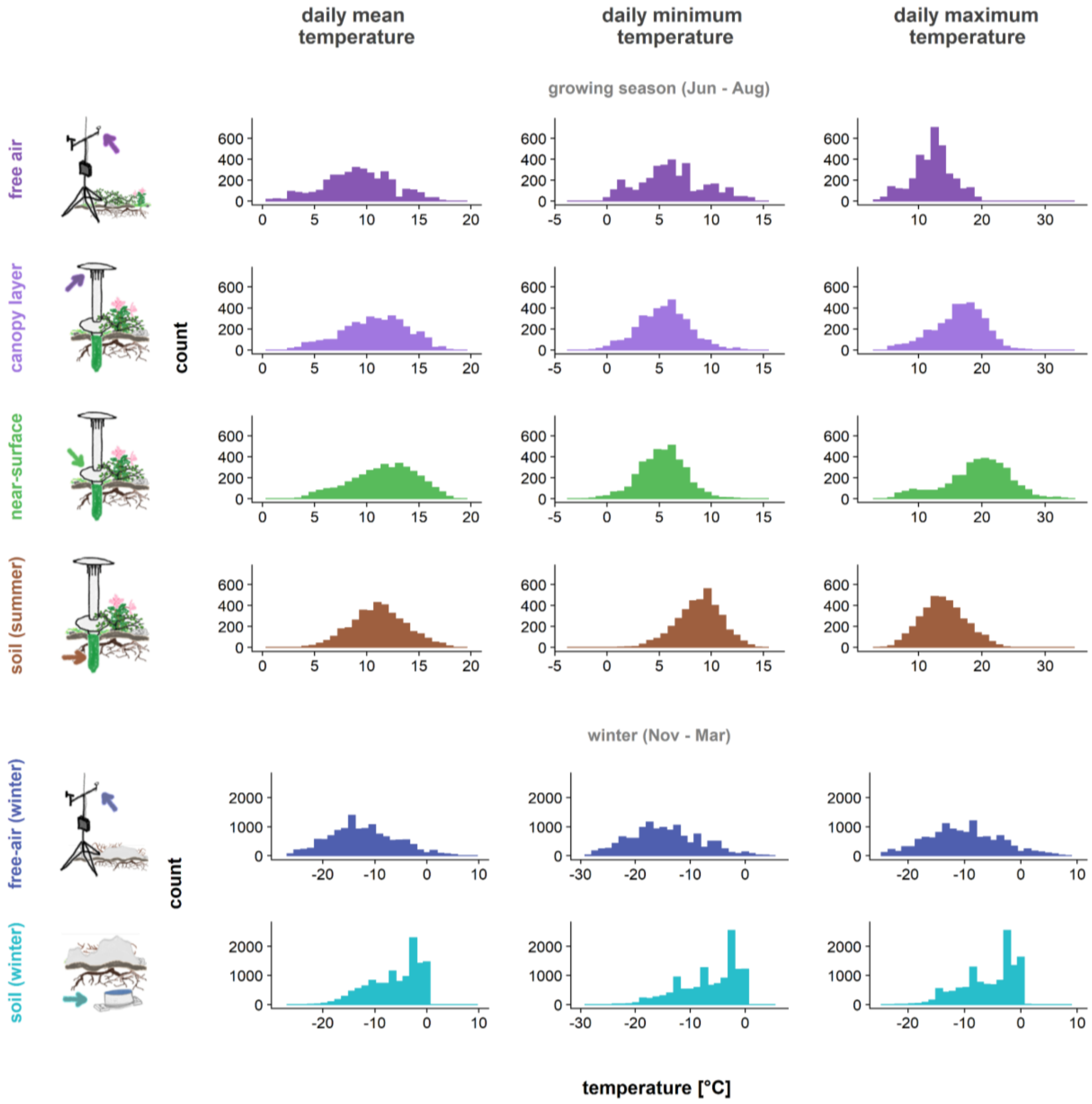

Figure S8:

Histograms of seasonal mean temperatures across the vegetation profile, per plot or weather station. Narrow distribution in weather station values is due to the low number of stations present and only a subset of these being highly correlated with air or soil temperatures in the plots (Figure S4).

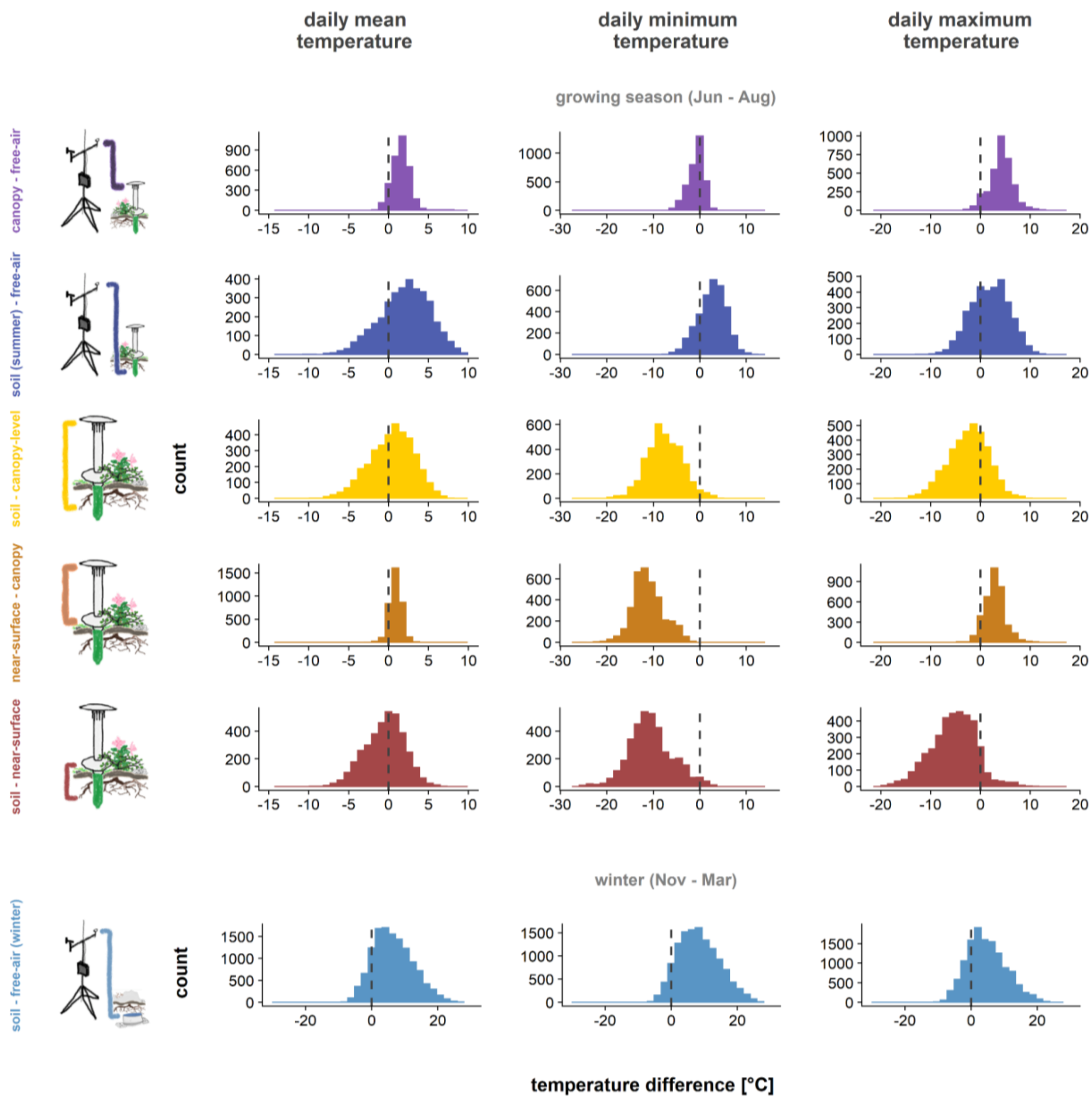

Figure S9:

Histograms of differences between daily mean, minimum and maximum temperatures in different layers of the vegetation profile. Negative values point to colder lower layers compared to higher elevated ones, while positive values indicate higher temperatures in lower layers. Note that vertical axis ranges vary between panels.

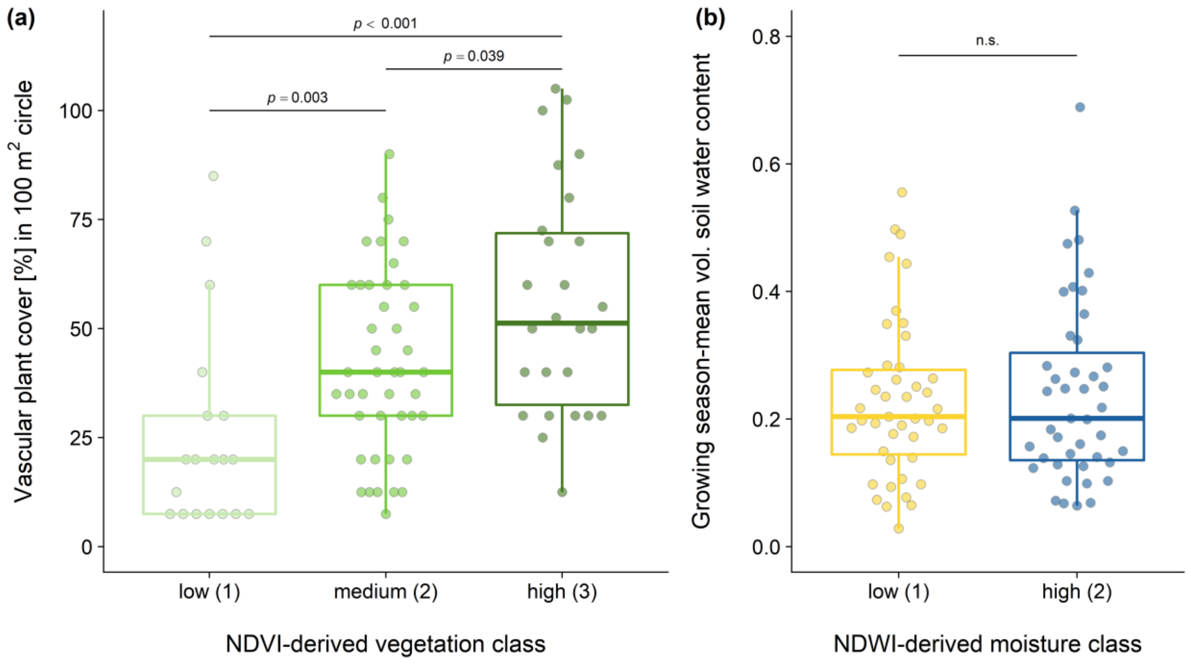

Figure S10:

The stratified-random sampling design succeeded to capture different vegetation cover, but not moisture conditions. (a) Cover values represent summed cover of shrubs, graminoids and forbs and could exceed 100 % in case of overlapping vegetation layers. **(b)** Soil moisture values were scaled across the range of daily mean values. P values were obtained from a Kruskal-Wallis test ($\chi^2 = 17.78$, df = 2) with post-hoc correction.

Supplementary tables

Table S1: Threshold values for the definition of vegetation and moisture classes. For vegetation classes, values are based on Normalised Difference Vegetation Index (NDVI), for moisture classes, values are based on Normalised Difference Wetness Index (NDWI). The thresholds were determined based on the distribution of values across the study area using k-means clustering.

**Vegetation**	**NDVI**	**Moisture**	**NDWI**
Low	> -0.21477<br>< 0.17910	Low	> -0.38214<br>< -0.02306
		High	> -0.02306<br>< 0.49108
Medium	> 0.17910<br>< 0.40434	Low	> -0.36011<br>< -0.02776
		High	> -0.02776<br>< 0.49430
High	> 0.40434<br>< 0.84541	Low	> -0.23849<br>< 0.09586
		High	> 0.09587<br>< 0.53435

Table S2: Pearson correlation coefficients for growing-season mean temperatures and growing degree days (GDD) for canopy-level, near-surface and soil sensors, and for winter (November - March) mean temperatures and freezing degree days (FDD) in the soil. GDD were calculated as the sum of daily mean temperatures above 0 (GDD0) and 5 °C (GDD5), respectively.

		canopy-level (growing season)			near-surface (growing season)			soil (growing season)			soil (Nov - Mar)	
		mean T	GDD0	GDD5	mean T	GDD0	GDD5	mean T	GDD0	GDD5	mean T	FDD
canopy-level (growing season)	mean T	--	0.71	0.71								
	GDD0	0.71	--	1.00								
	GDD5	0.71	1.00	--								
near-surface (growing season)	mean T				--	0.73	0.73					
	GDD0				0.73	--	1.00					
	GDD5				0.73	1.00	--					
soil (growing season)	mean T							--	0.93	0.93		
	GDD0							0.93	--	1.00		
	GDD5							0.93	1.00	--		
soil (Nov - Mar)	mean T										--	-1.00
	FDD										-1.00	--

Table S3: Pearson correlation coefficients for differences between growing-season mean temperatures and growing degree days (GDD) across vegetation layers, respectively, and between winter (November - March) mean temperatures and freezing degree days (FDD) in the soil and free air. GDD were calculated as the sum of daily mean temperatures above 0 (GDD0) and 5 °C (GDD5), respectively.

		canopy-level - free-air (growing season)			soil - free-air (growing season)			soil - canopy-level (growing season)			near-surface - canopy-level (growing season)			soil - near-surface (growing season)			soil - free-air (Nov - Mar)	
		mean T	GDD0	GDD5	mean T	GDD0	GDD5	mean T	GDD0	GDD5	mean T	GDD0	GDD5	mean T	GDD0	GDD5	mean T	FDD
canopy-level - free-air (growing season)	mean T	--	0.99	0.97														
	GDD0	0.99	--	0.99														
	GDD5	0.97	0.99	--														
soil - free-air (growing season)	mean T				--	0.99	0.99											
	GDD0				0.99	--	1.00											
	GDD5				0.99	1.00	--											
soil - canopy-level (growing season)	mean T							--	1.00	0.99								
	GDD0							1.00	--	1.00								
	GDD5							0.99	1.00	--								
near-surface - canopy-level (growing season)	mean T										--	0.99	0.98					
	GDD0										0.99	--	0.98					
	GDD5										0.98	0.98	--					
soil - near-surface (growing season)	mean T													--	1.00	0.99		
	GDD0													1.00	--	1.00		
	GDD5													0.99	1.00	--		
soil - free-air (Nov - Mar)	mean T																--	-1.00
	FDD																-1.00	--

Table S4: Predictor variables included in the different models of temperature responses across both seasons. Groups include temperature / temperature difference responses across all vegetation layers. Cover variables were included at their respective sampling resolution (plot radius 0.50, 2.00 or 5.64 m) into cross-scale models of growing-season temperature differences. See Table S5 for other predictors removed to avoid collinearity.

Temperature response group	Model formula
Growing-season daily [mean/min/max] temperature	temperature ~ daily free-air temperature + elevation + slope angle + solar radiation + landform + soil moisture + rock cover + shrub cover + bryophyte cover + lichen cover + average vegetation height + (1|plot ID)
Growing-season daily difference in [mean/min/max] temperature	temperature difference ~ daily free-air temperature + elevation + slope angle + solar radiation + landform + soil moisture + rock cover + shrub cover + bryophyte cover + lichen cover + average vegetation height + (1|plot ID)
Winter daily [mean/min/max] temperature	temperature ~ daily free-air temperature + elevation + slope angle + solar radiation + landform + snow cover duration + rock cover + shrub cover + average vegetation height + (1|plot ID)
Winter daily difference in [mean/min/max] temperature	temperature difference ~ daily free-air temperature + elevation + slope angle + solar radiation + landform + snow cover duration + rock cover + shrub cover + average vegetation height + (1|plot ID)

Table S5: Pearson correlation coefficients between all potential predictor variables for the small circles (radius 0.5 m). Either growing-season solar radiation, soil moisture, bryophyte and lichen cover (*, growing season) or snow cover duration and winter solar radiation (#, winter) were included as season-specific predictors (see also Table S4 for predictors included into each model). Shaded variables were removed prior to analyses to avoid collinearity with remaining variables. For clarity, only correlations with |*r*| > 0.3 are shown.

	elevation	slope inclination	solar rad. (SRI, growing season)	solar rad. (SRI, Nov – Mar)	landform	soil moisture	snow cover duration	rock cover	bare ground cover	shrub cover	bryophyte cover	lichen cover	litter cover	average vegetation height	average veg. height at logger	TMS top shield cover	TMS bottom shield cover	ground cover at logger
elevation	--							0.49		-0.44		-0.32	-0.30		-0.42			
slope inclination		--	-0.35	-0.85		-0.3				0.31	-0.38		0.30					
solar radiation (SRI, growing season) *		-0.35	--															
solar radiation (SRI, winter) #		-0.85		--							0.35		-0.34					
landform					--													
soil moisture		-0.30				--												
snow cover duration #							--		-0.32									
rock cover	0.49							--		-0.42								
bare ground cover							-0.32		--	-0.71			-0.43	-0.34	-0.43		-0.41	-0.48
shrub cover	-0.44	0.31						-0.42	-0.71	--			0.63	0.50	0.57		0.53	0.64
bryophyte cover *		-0.38		0.35							--							
lichen cover *	-0.32											--						
litter cover	-0.30	0.30		-0.34					-0.43	0.63			--	0.57	0.56	0.32	0.58	0.39
average vegetation height									-0.34	0.50			0.57	--	0.81	0.82	0.75	0.38
average veg. height at logger	-0.42								-0.43	0.57			0.56	0.81	--	0.62	0.74	0.45
TMS logger top shield cover													0.32	0.82	0.62	--	0.59	
TMS logger bottom shield cover									-0.41	0.53			0.58	0.75	0.74	0.59	--	0.54
ground cover at logger									-0.48	0.64			0.39	0.38	0.45		0.54	--

Table S6: Effect sizes (scaled & centred, backscaled, and converted into °C per meaningful units) for predictor variables for all models. One unit corresponds to the following respective difference for each predictor variable: 100m elevation; 1° slope angle; 1% relative solar radiation; 1 step on the categorical landform scale; 10% vol. soil moisture; 10 days of snow cover; 10% cover of rocks, shrubs, bryophytes or lichen; 1cm vegetation height.

model response	circle radius	temp. variable	predictor	post. 2.5% quantile	post. mean	post. 97.5% quantile	post. 2.5% quantile (backscaled)	post. mean (backscaled)	post. 97.5% quantile (backscaled)	post. 2.5% quantile (°C per unit)	post. mean (°C per unit)	post. 97.5% quantile (°C per unit)
canopy-layer temp.	50	mean	Intercept	10.599	10.668	10.738						
			elevation	-0.290	-0.191	-0.092	-68.725	-45.365	-21.880	-0.122	-0.081	-0.039
			slope angle	-0.015	0.083	0.178	-0.113	0.616	1.320	-0.002	0.011	0.024
			solar radiation	0.014	0.088	0.167	0.001	0.005	0.009	0.003	0.016	0.031
			landform	-0.019	0.048	0.120	-0.017	0.042	0.104	-0.022	0.055	0.138
			soil moisture	-0.164	-0.083	0.005	-0.016	-0.008	0.000	-0.170	-0.086	0.005
			rock cover	0.107	0.205	0.305	1.878	3.617	5.379	0.060	0.116	0.173
			shrub cover	-0.038	0.066	0.178	-0.959	1.678	4.534	-0.015	0.026	0.070
			bryophyte cover	-0.137	-0.054	0.021	-2.749	-1.083	0.416	-0.069	-0.027	0.010
			lichen cover	0.040	0.117	0.203	0.579	1.687	2.913	0.028	0.082	0.141
			vegetation height	0.015	0.099	0.185	0.039	0.266	0.496	0.005	0.037	0.069
canopy-layer temp.	50	min	Intercept	5.484	5.576	5.670						
			elevation	-0.275	-0.131	-0.003	-65.316	-31.097	-0.616	-0.116	-0.055	-0.001
			slope angle	0.028	0.163	0.297	0.209	1.211	2.205	0.004	0.022	0.040
			solar radiation	0.043	0.157	0.259	0.002	0.009	0.014	0.008	0.029	0.048
			landform	-0.008	0.088	0.183	-0.007	0.077	0.159	-0.009	0.102	0.210
			soil moisture	-0.258	-0.140	-0.025	-0.025	-0.014	-0.002	-0.267	-0.145	-0.026
			rock cover	0.024	0.171	0.307	0.423	3.011	5.404	0.014	0.097	0.174
			shrub cover	-0.259	-0.112	0.037	-6.601	-2.847	0.931	-0.101	-0.044	0.014
			bryophyte cover	-0.122	-0.017	0.094	-2.443	-0.331	1.885	-0.061	-0.008	0.047
			lichen cover	0.044	0.155	0.265	0.626	2.229	3.815	0.030	0.108	0.184
			vegetation height	-0.217	-0.104	0.017	-0.582	-0.279	0.045	-0.081	-0.039	0.006
canopy-layer temp.	50	max	Intercept	16.105	16.255	16.415						
			elevation	-0.595	-0.365	-0.156	-141.338	-86.731	-36.971	-0.251	-0.154	-0.066
			slope angle	0.143	0.350	0.561	1.062	2.597	4.164	0.019	0.047	0.075

model response	circle radius	temp. variable	predictor	post. 2.5% quantile	post. mean	post. 97.5% quantile	post. 2.5% quantile (backscaled)	post. mean (backscaled)	post. 97.5% quantile (backscaled)	post. 2.5% quantile (°C per unit)	post. mean (°C per unit)	post. 97.5% quantile (°C per unit)
canopy-layer temp. (cont.)	50	max	solar radiation	-0.242	-0.070	0.106	-0.013	-0.004	0.006	-0.045	-0.013	0.020
			landform	-0.179	-0.019	0.135	-0.156	-0.017	0.117	-0.206	-0.022	0.155
			soil moisture	-0.330	-0.148	0.044	-0.032	-0.014	0.004	-0.342	-0.153	0.046
			rock cover	-0.042	0.183	0.408	-0.736	3.222	7.197	-0.024	0.104	0.232
			shrub cover	0.024	0.256	0.473	0.603	6.536	12.062	0.009	0.100	0.185
			bryophyte cover	-0.310	-0.136	0.033	-6.200	-2.720	0.659	-0.155	-0.068	0.016
			lichen cover	-0.194	-0.002	0.173	-2.796	-0.036	2.482	-0.135	-0.002	0.120
			vegetation height	0.275	0.460	0.652	0.737	1.234	1.750	0.102	0.171	0.243
near-surface temp.	50	mean	Intercept	11.405	11.525	11.635						
			elevation	-0.257	-0.087	0.078	-60.918	-20.561	18.432	-0.108	-0.036	0.033
			slope angle	-0.046	0.125	0.280	-0.339	0.932	2.077	-0.006	0.017	0.038
			solar radiation	0.087	0.215	0.349	0.005	0.012	0.019	0.016	0.040	0.065
			landform	-0.059	0.062	0.180	-0.051	0.054	0.156	-0.068	0.072	0.207
			soil moisture	-0.300	-0.156	-0.014	-0.029	-0.015	-0.001	-0.311	-0.161	-0.014
			rock cover	0.030	0.202	0.390	0.524	3.560	6.873	0.017	0.115	0.221
			shrub cover	-0.099	0.074	0.253	-2.515	1.891	6.457	-0.039	0.029	0.099
			bryophyte cover	-0.366	-0.227	-0.100	-7.333	-4.552	-2.002	-0.183	-0.114	-0.050
			lichen cover	-0.069	0.078	0.210	-0.988	1.124	3.015	-0.048	0.054	0.146
			vegetation height	-0.211	-0.070	0.079	-0.565	-0.189	0.213	-0.078	-0.026	0.030
near-surface temp.	50	min	Intercept	5.327	5.455	5.576						
			elevation	-0.435	-0.253	-0.079	-103.222	-60.141	-18.782	-0.183	-0.107	-0.033
			slope angle	-0.003	0.161	0.335	-0.022	1.193	2.485	0.000	0.022	0.045
			solar radiation	-0.033	0.106	0.241	-0.002	0.006	0.013	-0.006	0.020	0.044
			landform	-0.019	0.107	0.233	-0.016	0.093	0.202	-0.022	0.123	0.268
			soil moisture	-0.138	0.002	0.172	-0.013	0.000	0.017	-0.143	0.002	0.178
			rock cover	0.176	0.362	0.544	3.097	6.371	9.592	0.100	0.205	0.309
			shrub cover	-0.355	-0.164	0.020	-9.061	-4.196	0.520	-0.139	-0.064	0.008
			bryophyte cover	-0.244	-0.099	0.044	-4.891	-1.990	0.889	-0.122	-0.050	0.022
			lichen cover	-0.064	0.093	0.237	-0.917	1.337	3.408	-0.044	0.065	0.165
			vegetation height	-0.194	-0.054	0.115	-0.521	-0.145	0.308	-0.072	-0.020	0.043

model response	circle radius	temp. variable	predictor	post. 2.5% quantile	post. mean	post. 97.5% quantile	post. 2.5% quantile (backscaled)	post. mean (backscaled)	post. 97.5% quantile (backscaled)	post. 2.5% quantile (°C per unit)	post. mean (°C per unit)	post. 97.5% quantile (°C per unit)
near-surface temp.	50	max	Intercept	18.813	19.091	19.434						
			elevation	-0.489	-0.038	0.401	-116.138	-8.919	95.152	-0.206	-0.016	0.169
			slope angle	0.128	0.546	0.967	0.948	4.056	7.181	0.017	0.074	0.130
			solar radiation	-0.038	0.326	0.655	-0.002	0.018	0.035	-0.007	0.060	0.121
			landform	-0.452	-0.133	0.170	-0.392	-0.115	0.147	-0.520	-0.153	0.195
			soil moisture	-0.917	-0.513	-0.164	-0.089	-0.050	-0.016	-0.950	-0.531	-0.170
			rock cover	-0.464	-0.014	0.464	-8.184	-0.249	8.171	-0.264	-0.008	0.263
			shrub cover	0.170	0.647	1.121	4.337	16.507	28.605	0.067	0.254	0.440
			bryophyte cover	-0.902	-0.524	-0.187	-18.042	-10.483	-3.739	-0.451	-0.262	-0.093
			lichen cover	-0.503	-0.156	0.219	-7.238	-2.250	3.153	-0.350	-0.109	0.152
			vegetation height	-0.358	0.025	0.397	-0.962	0.066	1.065	-0.134	0.009	0.148
soil temp. (growing season)	50	mean	Intercept	10.890	11.162	11.437						
			elevation	-0.166	0.200	0.616	-39.457	47.582	146.125	-0.070	0.084	0.259
			slope angle	-0.096	0.276	0.665	-0.717	2.047	4.938	-0.013	0.037	0.090
			solar radiation	0.023	0.358	0.658	0.001	0.019	0.036	0.004	0.066	0.122
			landform	-0.034	0.256	0.517	-0.029	0.222	0.449	-0.039	0.295	0.595
			soil moisture	-0.383	-0.047	0.280	-0.037	-0.004	0.027	-0.397	-0.048	0.290
			rock cover	-0.178	0.234	0.648	-3.132	4.121	11.411	-0.101	0.133	0.367
			shrub cover	-0.982	-0.587	-0.142	-25.038	-14.971	-3.627	-0.385	-0.230	-0.056
			bryophyte cover	-0.987	-0.702	-0.352	-19.752	-14.043	-7.036	-0.493	-0.351	-0.176
			lichen cover	-0.449	-0.146	0.178	-6.461	-2.097	2.561	-0.312	-0.101	0.124
			vegetation height	-0.797	-0.453	-0.118	-2.139	-1.215	-0.317	-0.297	-0.169	-0.044
soil temp. (growing season)	50	min	Intercept	8.648	8.893	9.117						
			elevation	-0.321	0.057	0.389	-76.281	13.640	92.368	-0.135	0.024	0.164
			slope angle	-0.141	0.188	0.538	-1.050	1.395	3.998	-0.019	0.025	0.072
			solar radiation	-0.032	0.224	0.509	-0.002	0.012	0.028	-0.006	0.041	0.094
			landform	-0.124	0.121	0.388	-0.108	0.105	0.337	-0.143	0.139	0.447
			soil moisture	-0.200	0.080	0.392	-0.019	0.008	0.038	-0.207	0.083	0.406
			rock cover	-0.093	0.301	0.675	-1.639	5.312	11.900	-0.053	0.171	0.383
			shrub cover	-0.468	-0.092	0.277	-11.935	-2.355	7.061	-0.183	-0.036	0.109

model response	circle radius	temp. variable	predictor	post. 2.5% quantile	post. mean	post. 97.5% quantile	post. 2.5% quantile (backscaled)	post. mean (backscaled)	post. 97.5% quantile (backscaled)	post. 2.5% quantile (°C per unit)	post. mean (°C per unit)	post. 97.5% quantile (°C per unit)
soil temp. (growing season) (cont.)	50	min	bryophyte cover	-0.877	-0.600	-0.312	-17.551	-11.999	-6.253	-0.438	-0.300	-0.156
			lichen cover	-0.223	0.070	0.368	-3.203	1.008	5.286	-0.155	0.049	0.256
			vegetation height	-0.711	-0.408	-0.111	-1.910	-1.095	-0.297	-0.265	-0.152	-0.041
soil temp. (growing season)	50	max	Intercept	13.700	14.074	14.451						
			elevation	-0.184	0.357	0.914	-43.563	84.675	216.996	-0.077	0.150	0.385
			slope angle	-0.049	0.438	0.966	-0.361	3.251	7.178	-0.007	0.059	0.130
			solar radiation	-0.049	0.373	0.793	-0.003	0.020	0.043	-0.009	0.069	0.147
			landform	0.023	0.414	0.799	0.020	0.359	0.694	0.026	0.476	0.920
			soil moisture	-0.662	-0.227	0.258	-0.064	-0.022	0.025	-0.686	-0.235	0.267
			rock cover	-0.508	0.023	0.597	-8.948	0.397	10.514	-0.288	0.013	0.339
			shrub cover	-1.667	-1.124	-0.500	-42.521	-28.681	-12.761	-0.654	-0.441	-0.196
			bryophyte cover	-1.339	-0.890	-0.484	-26.786	-17.815	-9.694	-0.669	-0.445	-0.242
			lichen cover	-0.931	-0.488	-0.062	-13.393	-7.019	-0.896	-0.647	-0.339	-0.043
			vegetation height	-0.979	-0.550	-0.044	-2.628	-1.477	-0.119	-0.365	-0.205	-0.017
soil temp. (winter)	50	mean	Intercept	-6.442	-6.006	-5.547						
			elevation	-1.143	-0.525	0.069	-271.266	-124.655	16.268	-0.482	-0.221	0.029
			solar radiation	-1.061	-0.506	-0.006	-0.068	-0.033	0.000	-0.165	-0.079	-0.001
			landform	-0.604	-0.103	0.383	-0.525	-0.089	0.333	-0.696	-0.119	0.441
			snow cover duration	2.508	2.985	3.554	95.317	113.466	135.094	0.660	0.785	0.935
			rock cover	-1.479	-0.925	-0.302	-26.059	-16.300	-5.320	-0.839	-0.525	-0.171
			shrub cover	-1.281	-0.626	0.009	-32.674	-15.966	0.230	-0.502	-0.245	0.004
			bryophyte cover	-0.899	-0.391	0.108	-17.988	-7.833	2.164	-0.449	-0.196	0.054
			lichen cover	-1.262	-0.701	-0.165	-18.151	-10.076	-2.375	-0.877	-0.487	-0.115
			vegetation height	-0.658	-0.129	0.413	-1.766	-0.346	1.109	-0.245	-0.048	0.154
soil temp. (winter)	50	min	Intercept	-6.927	-6.434	-5.995						
			elevation	-1.171	-0.532	0.115	-277.887	-126.202	27.260	-0.493	-0.224	0.048
			solar radiation	-1.060	-0.541	0.020	-0.068	-0.035	0.001	-0.164	-0.084	0.003
			landform	-0.601	-0.101	0.394	-0.522	-0.088	0.342	-0.692	-0.116	0.454
			snow cover duration	2.895	3.405	3.943	110.025	129.430	149.871	0.761	0.896	1.037

model response	circle radius	temp. variable	predictor	post. 2.5% quantile	post. mean	post. 97.5% quantile	post. 2.5% quantile (backscaled)	post. mean (backscaled)	post. 97.5% quantile (backscaled)	post. 2.5% quantile (°C per unit)	post. mean (°C per unit)	post. 97.5% quantile (°C per unit)
soil temp. (winter) (cont.)	50	min	rock cover	-1.591	-0.952	-0.344	-28.032	-16.779	-6.062	-0.903	-0.540	-0.195
			shrub cover	-1.293	-0.641	0.042	-32.973	-16.348	1.076	-0.507	-0.251	0.017
			bryophyte cover	-0.936	-0.391	0.102	-18.725	-7.821	2.049	-0.468	-0.195	0.051
			lichen cover	-1.284	-0.709	-0.137	-18.475	-10.201	-1.968	-0.893	-0.493	-0.095
			vegetation height	-0.711	-0.143	0.401	-1.908	-0.383	1.076	-0.265	-0.053	0.149
soil temp. (winter)	50	max	Intercept	-6.024	-5.605	-5.186						
			elevation	-1.095	-0.519	0.051	-259.937	-123.256	12.046	-0.461	-0.219	0.021
			solar radiation	-0.953	-0.479	0.069	-0.061	-0.031	0.004	-0.148	-0.074	0.011
			landform	-0.589	-0.104	0.355	-0.512	-0.090	0.309	-0.679	-0.119	0.409
			snow cover duration	2.107	2.585	3.149	80.072	98.271	119.716	0.554	0.680	0.829
			rock cover	-1.496	-0.915	-0.352	-26.361	-16.132	-6.202	-0.849	-0.520	-0.200
			shrub cover	-1.260	-0.615	-0.027	-32.133	-15.689	-0.690	-0.494	-0.241	-0.011
			bryophyte cover	-0.881	-0.391	0.129	-17.623	-7.819	2.577	-0.440	-0.195	0.064
			lichen cover	-1.247	-0.699	-0.152	-17.943	-10.055	-2.185	-0.867	-0.486	-0.106
			vegetation height	-0.643	-0.114	0.434	-1.725	-0.306	1.165	-0.239	-0.042	0.162
soil - canopy T difference	50	mean	Intercept	0.221	0.486	0.731						
			elevation	-0.038	0.343	0.705	-8.984	81.464	167.436	-0.016	0.145	0.297
			slope angle	-0.118	0.252	0.605	-0.879	1.873	4.493	-0.016	0.034	0.081
			solar radiation	-0.009	0.282	0.567	-0.001	0.015	0.031	-0.002	0.052	0.105
			landform	-0.023	0.222	0.495	-0.020	0.193	0.430	-0.026	0.256	0.570
			soil moisture	-0.257	0.046	0.381	-0.025	0.004	0.037	-0.266	0.047	0.394
			rock cover	-0.403	0.005	0.387	-7.099	0.081	6.819	-0.229	0.003	0.220
			shrub cover	-1.092	-0.693	-0.278	-27.854	-17.674	-7.088	-0.428	-0.272	-0.109
			bryophyte cover	-0.928	-0.639	-0.323	-18.571	-12.792	-6.456	-0.464	-0.319	-0.161
			lichen cover	-0.630	-0.318	-0.004	-9.064	-4.574	-0.056	-0.438	-0.221	-0.003
			vegetation height	-0.876	-0.561	-0.240	-2.352	-1.505	-0.644	-0.326	-0.209	-0.089
soil - canopy T difference	50	min	Intercept	-7.627	-7.364	-7.098						
			elevation	0.018	0.392	0.763	4.340	92.965	181.169	0.008	0.165	0.322
			slope angle	-0.485	-0.111	0.257	-3.600	-0.827	1.906	-0.065	-0.015	0.035
			solar radiation	0.031	0.304	0.594	0.002	0.016	0.032	0.006	0.056	0.110

model response	circle radius	temp. variable	predictor	post. 2.5% quantile	post. mean	post. 97.5% quantile	post. 2.5% quantile (backscaled)	post. mean (backscaled)	post. 97.5% quantile (backscaled)	post. 2.5% quantile (°C per unit)	post. mean (°C per unit)	post. 97.5% quantile (°C per unit)
soil - canopy T difference (cont.)	50	min	landform	-0.122	0.154	0.421	-0.106	0.134	0.366	-0.141	0.177	0.485
			soil moisture	-0.094	0.235	0.537	-0.009	0.023	0.052	-0.097	0.243	0.556
			rock cover	-0.309	0.096	0.502	-5.444	1.699	8.852	-0.175	0.055	0.285
			shrub cover	-0.780	-0.365	0.032	-19.908	-9.315	0.810	-0.306	-0.143	0.012
			bryophyte cover	-0.745	-0.461	-0.150	-14.914	-9.227	-3.011	-0.372	-0.230	-0.075
			lichen cover	-0.290	0.021	0.318	-4.175	0.295	4.576	-0.202	0.014	0.221
			vegetation height	-1.198	-0.886	-0.556	-3.216	-2.379	-1.494	-0.446	-0.330	-0.207
soil - canopy T difference	50	max	Intercept	-2.594	-2.208	-1.809						
			elevation	0.064	0.639	1.181	15.137	151.654	280.360	0.027	0.269	0.498
			slope angle	-0.377	0.136	0.721	-2.799	1.008	5.354	-0.051	0.018	0.097
			solar radiation	0.009	0.465	0.914	0.000	0.025	0.049	0.002	0.086	0.169
			landform	0.053	0.455	0.863	0.046	0.395	0.750	0.061	0.524	0.994
			soil moisture	-0.548	-0.082	0.409	-0.053	-0.008	0.040	-0.568	-0.085	0.424
			rock cover	-0.725	-0.161	0.435	-12.778	-2.830	7.668	-0.412	-0.091	0.247
			shrub cover	-2.081	-1.443	-0.854	-53.088	-36.795	-21.791	-0.816	-0.566	-0.335
			bryophyte cover	-1.200	-0.750	-0.310	-24.013	-15.016	-6.212	-0.600	-0.375	-0.155
			lichen cover	-0.998	-0.541	-0.055	-14.360	-7.782	-0.792	-0.694	-0.376	-0.038
			vegetation height	-1.493	-1.010	-0.522	-4.009	-2.712	-1.401	-0.556	-0.376	-0.194
near-surface - canopy T difference	50	mean	Intercept	0.748	0.842	0.935						
			elevation	-0.022	0.104	0.238	-5.230	24.573	56.519	-0.009	0.044	0.100
			slope angle	-0.074	0.056	0.184	-0.549	0.418	1.364	-0.010	0.008	0.025
			solar radiation	0.023	0.129	0.226	0.001	0.007	0.012	0.004	0.024	0.042
			landform	-0.078	0.017	0.119	-0.068	0.015	0.103	-0.090	0.019	0.137
			soil moisture	-0.176	-0.070	0.041	-0.017	-0.007	0.004	-0.183	-0.072	0.042
			rock cover	-0.135	0.003	0.141	-2.371	0.049	2.487	-0.076	0.002	0.080
			shrub cover	-0.143	-0.003	0.135	-3.653	-0.087	3.449	-0.056	-0.001	0.053
			bryophyte cover	-0.261	-0.158	-0.045	-5.224	-3.157	-0.899	-0.130	-0.079	-0.022
			lichen cover	-0.145	-0.037	0.077	-2.088	-0.525	1.114	-0.101	-0.025	0.054
			vegetation height	-0.281	-0.161	-0.055	-0.753	-0.433	-0.147	-0.105	-0.060	-0.020

model response	circle radius	temp. variable	predictor	post. 2.5% quantile	post. mean	post. 97.5% quantile	post. 2.5% quantile (backscaled)	post. mean (backscaled)	post. 97.5% quantile (backscaled)	post. 2.5% quantile (°C per unit)	post. mean (°C per unit)	post. 97.5% quantile (°C per unit)
near-surface - canopy T difference	50	min	Intercept	-10.966	-10.802	-10.601						
			elevation	-0.194	0.095	0.351	-45.997	22.548	83.255	-0.082	0.040	0.148
			slope angle	-0.433	-0.166	0.092	-3.219	-1.235	0.684	-0.058	-0.022	0.012
			solar radiation	-0.032	0.179	0.386	-0.002	0.010	0.021	-0.006	0.033	0.071
			landform	-0.058	0.135	0.318	-0.050	0.117	0.276	-0.067	0.155	0.366
			soil moisture	-0.073	0.150	0.373	-0.007	0.014	0.036	-0.075	0.155	0.386
			rock cover	-0.119	0.168	0.433	-2.094	2.960	7.635	-0.067	0.095	0.246
			shrub cover	-0.741	-0.440	-0.162	-18.891	-11.229	-4.138	-0.290	-0.173	-0.064
			bryophyte cover	-0.163	0.045	0.258	-3.256	0.900	5.173	-0.081	0.022	0.129
			lichen cover	-0.161	0.079	0.285	-2.317	1.133	4.094	-0.112	0.055	0.198
			vegetation height	-0.739	-0.516	-0.287	-1.983	-1.385	-0.772	-0.275	-0.192	-0.107
near-surface - canopy T difference	50	max	Intercept	2.533	2.779	3.024						
			elevation	-0.014	0.330	0.704	-3.296	78.363	167.159	-0.006	0.139	0.297
			slope angle	-0.125	0.214	0.564	-0.929	1.591	4.186	-0.017	0.029	0.076
			solar radiation	0.106	0.383	0.654	0.006	0.021	0.035	0.020	0.071	0.121
			landform	-0.361	-0.110	0.151	-0.314	-0.096	0.131	-0.416	-0.127	0.174
			soil moisture	-0.641	-0.357	-0.067	-0.062	-0.035	-0.006	-0.664	-0.370	-0.069
			rock cover	-0.546	-0.169	0.210	-9.619	-2.972	3.697	-0.310	-0.096	0.119
			shrub cover	-0.035	0.333	0.720	-0.904	8.485	18.365	-0.014	0.130	0.282
			bryophyte cover	-0.615	-0.345	-0.064	-12.315	-6.909	-1.289	-0.308	-0.173	-0.032
			lichen cover	-0.408	-0.113	0.163	-5.874	-1.624	2.349	-0.284	-0.079	0.114
			vegetation height	-0.677	-0.392	-0.083	-1.817	-1.051	-0.222	-0.252	-0.146	-0.031
soil - near-surface T difference	50	mean	Intercept	-0.571	-0.367	-0.137						
			elevation	-0.018	0.283	0.612	-4.226	67.188	145.152	-0.008	0.119	0.258
			slope angle	-0.126	0.159	0.469	-0.933	1.178	3.481	-0.017	0.021	0.063
			solar radiation	-0.099	0.144	0.384	-0.005	0.008	0.021	-0.018	0.027	0.071
			landform	-0.049	0.195	0.401	-0.043	0.169	0.348	-0.057	0.225	0.462
			soil moisture	-0.154	0.112	0.384	-0.015	0.011	0.037	-0.160	0.116	0.397
			rock cover	-0.282	0.027	0.352	-4.962	0.479	6.210	-0.160	0.015	0.200
			shrub cover	-1.026	-0.672	-0.352	-26.167	-17.146	-8.973	-0.402	-0.264	-0.138

model response	circle radius	temp. variable	predictor	post. 2.5% quantile	post. mean	post. 97.5% quantile	post. 2.5% quantile (backscaled)	post. mean (backscaled)	post. 97.5% quantile (backscaled)	post. 2.5% quantile (°C per unit)	post. mean (°C per unit)	post. 97.5% quantile (°C per unit)
soil - near-surface T difference (cont.)	50	mean	bryophyte cover	-0.719	-0.474	-0.221	-14.390	-9.495	-4.430	-0.359	-0.237	-0.111
			lichen cover	-0.481	-0.230	0.042	-6.919	-3.301	0.606	-0.334	-0.160	0.029
			vegetation height	-0.681	-0.385	-0.133	-1.828	-1.035	-0.356	-0.254	-0.144	-0.049
soil - near-surface T difference	50	min	Intercept	-10.542	-10.199	-9.866						
			elevation	-0.377	0.104	0.598	-89.444	24.732	142.048	-0.159	0.044	0.252
			slope angle	-0.858	-0.354	0.075	-6.374	-2.632	0.557	-0.116	-0.048	0.010
			solar radiation	-0.462	-0.093	0.320	-0.025	-0.005	0.017	-0.085	-0.017	0.059
			landform	-0.095	0.259	0.592	-0.083	0.225	0.514	-0.110	0.298	0.681
			soil moisture	0.168	0.587	1.001	0.016	0.057	0.097	0.174	0.608	1.036
			rock cover	-0.233	0.321	0.832	-4.107	5.651	14.658	-0.132	0.182	0.472
			shrub cover	-1.250	-0.730	-0.184	-31.892	-18.618	-4.695	-0.490	-0.286	-0.072
			bryophyte cover	-0.468	-0.066	0.316	-9.372	-1.321	6.331	-0.234	-0.033	0.158
			lichen cover	-0.153	0.233	0.644	-2.200	3.346	9.259	-0.106	0.162	0.448
			vegetation height	-0.839	-0.436	0.009	-2.251	-1.171	0.023	-0.312	-0.163	0.003
soil - near-surface T difference	50	max	Intercept	-5.450	-5.020	-4.600						
			elevation	-0.200	0.398	1.023	-47.525	94.424	242.760	-0.084	0.168	0.431
			slope angle	-0.724	-0.114	0.465	-5.381	-0.848	3.452	-0.098	-0.015	0.063
			solar radiation	-0.410	0.051	0.528	-0.022	0.003	0.029	-0.076	0.009	0.098
			landform	0.100	0.547	0.976	0.086	0.475	0.848	0.115	0.630	1.124
			soil moisture	-0.202	0.286	0.846	-0.020	0.028	0.082	-0.210	0.296	0.876
			rock cover	-0.574	0.048	0.697	-10.120	0.850	12.275	-0.326	0.027	0.395
			shrub cover	-2.389	-1.759	-1.126	-60.943	-44.879	-28.708	-0.937	-0.690	-0.441
			bryophyte cover	-0.896	-0.364	0.074	-17.932	-7.278	1.478	-0.448	-0.182	0.037
			lichen cover	-0.816	-0.328	0.162	-11.737	-4.712	2.327	-0.567	-0.228	0.112
			vegetation height	-1.046	-0.578	-0.021	-2.809	-1.553	-0.055	-0.390	-0.215	-0.008
soil - free-air T difference (winter)	50	mean	Intercept	6.127	6.615	7.075						
			elevation	0.201	0.857	1.516	47.628	203.517	359.743	0.085	0.361	0.639
			solar radiation	-0.977	-0.413	0.123	-0.063	-0.027	0.008	-0.152	-0.064	0.019

model response	circle radius	temp. variable	predictor	post. 2.5% quantile	post. mean	post. 97.5% quantile	post. 2.5% quantile (backscaled)	post. mean (backscaled)	post. 97.5% quantile (backscaled)	post. 2.5% quantile (°C per unit)	post. mean (°C per unit)	post. 97.5% quantile (°C per unit)
soil - free-air T difference (winter) (cont.)	50	mean	landform	-0.542	-0.052	0.464	-0.471	-0.045	0.403	-0.624	-0.060	0.534
			snow cover duration	2.658	3.203	3.736	101.050	121.757	141.993	0.699	0.843	0.983
			rock cover	-1.560	-0.943	-0.331	-27.493	-16.611	-5.840	-0.885	-0.535	-0.188
			shrub cover	-1.582	-0.917	-0.255	-40.348	-23.387	-6.511	-0.620	-0.359	-0.100
			bryophyte cover	-0.822	-0.277	0.273	-16.457	-5.548	5.473	-0.411	-0.139	0.137
			lichen cover	-1.408	-0.823	-0.218	-20.246	-11.844	-3.143	-0.979	-0.573	-0.152
			vegetation height	-0.735	-0.159	0.388	-1.972	-0.427	1.043	-0.274	-0.059	0.145
soil - free-air T difference (winter)	50	min	Intercept	7.949	8.483	9.020						
			elevation	-0.112	0.563	1.287	-26.615	133.657	305.507	-0.047	0.237	0.542
			solar radiation	-0.938	-0.350	0.249	-0.060	-0.023	0.016	-0.145	-0.054	0.039
			landform	-0.587	-0.037	0.541	-0.509	-0.032	0.470	-0.675	-0.042	0.623
			snow cover duration	2.994	3.630	4.207	113.807	137.989	159.901	0.788	0.955	1.107
			rock cover	-1.651	-0.982	-0.306	-29.085	-17.304	-5.398	-0.937	-0.557	-0.174
			shrub cover	-1.707	-1.017	-0.254	-43.529	-25.949	-6.485	-0.669	-0.399	-0.100
			bryophyte cover	-0.858	-0.274	0.317	-17.178	-5.483	6.339	-0.429	-0.137	0.158
			lichen cover	-1.548	-0.838	-0.242	-22.258	-12.052	-3.475	-1.076	-0.583	-0.168
			vegetation height	-0.849	-0.204	0.413	-2.280	-0.548	1.107	-0.316	-0.076	0.154
soil - free-air T difference (winter)	50	max	Intercept	4.367	4.829	5.295						
			elevation	0.491	1.051	1.682	116.610	249.336	399.132	0.207	0.443	0.709
			solar radiation	-0.919	-0.422	0.094	-0.059	-0.027	0.006	-0.143	-0.065	0.015
			landform	-0.552	-0.063	0.389	-0.479	-0.054	0.338	-0.636	-0.072	0.448
			snow cover duration	2.300	2.805	3.319	87.421	106.630	126.177	0.605	0.738	0.873
			rock cover	-1.513	-0.908	-0.348	-26.656	-16.004	-6.127	-0.858	-0.515	-0.197
			shrub cover	-1.498	-0.856	-0.244	-38.212	-21.841	-6.230	-0.587	-0.336	-0.096
			bryophyte cover	-0.783	-0.293	0.234	-15.677	-5.862	4.684	-0.391	-0.146	0.117
			lichen cover	-1.434	-0.837	-0.315	-20.623	-12.044	-4.529	-0.997	-0.582	-0.219
			vegetation height	-0.704	-0.130	0.365	-1.889	-0.348	0.979	-0.262	-0.048	0.136

model response	circle radius	temp. variable	predictor	post. 2.5% quantile	post. mean	post. 97.5% quantile	post. 2.5% quantile (backscaled)	post. mean (backscaled)	post. 97.5% quantile (backscaled)	post. 2.5% quantile (°C per unit)	post. mean (°C per unit)	post. 97.5% quantile (°C per unit)
canopy - free-air T difference	50	mean	Intercept	1.457	1.548	1.631						
			elevation	0.168	0.305	0.421	39.834	72.331	99.998	0.071	0.128	0.178
			slope angle	-0.038	0.085	0.210	-0.282	0.633	1.557	-0.005	0.011	0.028
			solar radiation	0.075	0.172	0.278	0.004	0.009	0.015	0.014	0.032	0.051
			landform	-0.105	-0.010	0.078	-0.091	-0.009	0.068	-0.121	-0.011	0.090
			soil moisture	-0.191	-0.082	0.021	-0.018	-0.008	0.002	-0.198	-0.085	0.022
			rock cover	0.047	0.188	0.315	0.836	3.308	5.551	0.027	0.107	0.179
			shrub cover	-0.098	0.032	0.168	-2.503	0.804	4.289	-0.038	0.012	0.066
			bryophyte cover	-0.137	-0.039	0.062	-2.743	-0.789	1.234	-0.069	-0.020	0.031
			lichen cover	-0.096	0.010	0.108	-1.388	0.150	1.548	-0.067	0.007	0.075
			vegetation height	-0.024	0.083	0.195	-0.063	0.222	0.523	-0.009	0.031	0.073
canopy - free-air T difference	50	min	Intercept	-0.696	-0.585	-0.482						
			elevation	-0.018	0.131	0.286	-4.378	31.049	67.822	-0.008	0.055	0.120
			slope angle	0.007	0.163	0.317	0.051	1.210	2.355	0.001	0.022	0.043
			solar radiation	0.058	0.179	0.306	0.003	0.010	0.017	0.011	0.033	0.057
			landform	-0.029	0.086	0.197	-0.026	0.074	0.171	-0.034	0.099	0.227
			soil moisture	-0.310	-0.176	-0.047	-0.030	-0.017	-0.005	-0.322	-0.182	-0.049
			rock cover	-0.061	0.098	0.263	-1.082	1.735	4.642	-0.035	0.056	0.149
			shrub cover	-0.406	-0.234	-0.071	-10.348	-5.972	-1.820	-0.159	-0.092	-0.028
			bryophyte cover	-0.133	-0.008	0.117	-2.662	-0.151	2.342	-0.066	-0.004	0.058
			lichen cover	-0.081	0.049	0.185	-1.171	0.703	2.656	-0.057	0.034	0.128
			vegetation height	-0.308	-0.167	-0.040	-0.827	-0.448	-0.107	-0.115	-0.062	-0.015
canopy - free-air T difference	50	max	Intercept	3.991	4.179	4.364						
			elevation	0.096	0.368	0.630	22.683	87.317	149.440	0.040	0.155	0.265
			slope angle	0.058	0.315	0.557	0.434	2.341	4.137	0.008	0.042	0.075
			solar radiation	-0.129	0.062	0.284	-0.007	0.003	0.015	-0.024	0.011	0.053
			landform	-0.295	-0.111	0.073	-0.257	-0.097	0.063	-0.340	-0.128	0.084
			soil moisture	-0.336	-0.115	0.113	-0.032	-0.011	0.011	-0.348	-0.119	0.117
			rock cover	-0.037	0.216	0.494	-0.648	3.810	8.697	-0.021	0.123	0.280
			shrub cover	0.007	0.289	0.574	0.167	7.363	14.639	0.003	0.113	0.225

model response	circle radius	temp. variable	predictor	post. 2.5% quantile	post. mean	post. 97.5% quantile	post. 2.5% quantile (backscaled)	post. mean (backscaled)	post. 97.5% quantile (backscaled)	post. 2.5% quantile (°C per unit)	post. mean (°C per unit)	post. 97.5% quantile (°C per unit)
canopy - free-air T difference (cont.)	50	max	bryophyte cover	-0.330	-0.120	0.090	-6.599	-2.409	1.804	-0.165	-0.060	0.045
			lichen cover	-0.314	-0.110	0.104	-4.510	-1.585	1.502	-0.218	-0.077	0.073
			vegetation height	0.254	0.479	0.702	0.682	1.287	1.883	0.095	0.179	0.261
soil - free-air T difference (growing season)	50	mean	Intercept	1.789	2.055	2.312						
			elevation	0.314	0.693	1.069	74.579	164.422	253.658	0.132	0.292	0.450
			slope angle	-0.049	0.302	0.659	-0.367	2.240	4.897	-0.007	0.041	0.089
			solar radiation	0.140	0.441	0.728	0.008	0.024	0.039	0.026	0.082	0.134
			landform	-0.060	0.207	0.478	-0.052	0.179	0.415	-0.069	0.238	0.551
			soil moisture	-0.347	-0.029	0.309	-0.033	-0.003	0.030	-0.359	-0.030	0.320
			rock cover	-0.188	0.201	0.605	-3.312	3.546	10.666	-0.107	0.114	0.344
			shrub cover	-1.018	-0.622	-0.222	-25.964	-15.854	-5.652	-0.399	-0.244	-0.087
			bryophyte cover	-0.996	-0.693	-0.388	-19.927	-13.874	-7.773	-0.498	-0.346	-0.194
			lichen cover	-0.568	-0.271	0.033	-8.173	-3.898	0.472	-0.395	-0.188	0.023
			vegetation height	-0.803	-0.477	-0.173	-2.155	-1.282	-0.464	-0.299	-0.178	-0.064
soil - free-air T difference (growing season)	50	min	Intercept	2.481	2.732	2.990						
			elevation	-0.067	0.325	0.680	-15.835	77.229	161.458	-0.028	0.137	0.287
			slope angle	-0.117	0.209	0.594	-0.868	1.551	4.414	-0.016	0.028	0.080
			solar radiation	-0.053	0.244	0.525	-0.003	0.013	0.028	-0.010	0.045	0.097
			landform	-0.114	0.123	0.411	-0.099	0.107	0.357	-0.132	0.142	0.473
			soil moisture	-0.235	0.060	0.376	-0.023	0.006	0.036	-0.244	0.062	0.389
			rock cover	-0.201	0.221	0.593	-3.547	3.898	10.450	-0.114	0.126	0.337
			shrub cover	-0.603	-0.209	0.195	-15.389	-5.325	4.970	-0.237	-0.082	0.076
			bryophyte cover	-0.900	-0.585	-0.308	-18.003	-11.714	-6.159	-0.450	-0.293	-0.154
			lichen cover	-0.320	-0.037	0.276	-4.600	-0.538	3.967	-0.222	-0.026	0.192
			vegetation height	-0.814	-0.480	-0.170	-2.185	-1.289	-0.458	-0.303	-0.179	-0.064
soil - free-air T difference (growing season)	50	max	Intercept	1.622	2.005	2.367						
			elevation	0.514	1.079	1.556	122.031	256.129	369.255	0.217	0.455	0.655
			slope angle	-0.063	0.433	0.919	-0.469	3.217	6.824	-0.009	0.058	0.124

model response	circle radius	temp. variable	predictor	post. 2.5% quantile	post. mean	post. 97.5% quantile	post. 2.5% quantile (backscaled)	post. mean (backscaled)	post. 97.5% quantile (backscaled)	post. 2.5% quantile (°C per unit)	post. mean (°C per unit)	post. 97.5% quantile (°C per unit)
soil - free-air T difference (growing season) (cont.)	50	max	solar radiation	0.130	0.512	0.935	0.007	0.028	0.051	0.024	0.095	0.173
			landform	-0.053	0.324	0.691	-0.046	0.282	0.600	-0.061	0.373	0.796
			soil moisture	-0.619	-0.182	0.268	-0.060	-0.018	0.026	-0.641	-0.188	0.278
			rock cover	-0.528	0.041	0.598	-9.304	0.714	10.537	-0.300	0.023	0.339
			shrub cover	-1.664	-1.081	-0.537	-42.442	-27.574	-13.709	-0.652	-0.424	-0.211
			bryophyte cover	-1.292	-0.877	-0.465	-25.846	-17.549	-9.300	-0.645	-0.438	-0.232
			lichen cover	-1.048	-0.619	-0.190	-15.069	-8.897	-2.738	-0.728	-0.430	-0.132
			vegetation height	-0.997	-0.544	-0.094	-2.677	-1.459	-0.253	-0.371	-0.202	-0.035
soil - canopy T difference	200	mean	Intercept	0.234	0.487	0.728						
			elevation	-0.028	0.311	0.641	-6.715	73.781	152.023	-0.012	0.131	0.270
			slope angle	-0.074	0.293	0.638	-0.547	2.174	4.736	-0.010	0.039	0.086
			solar radiation	0.091	0.373	0.675	0.005	0.020	0.037	0.017	0.069	0.125
			landform	-0.096	0.181	0.418	-0.083	0.157	0.363	-0.110	0.209	0.481
			soil moisture	-0.167	0.132	0.447	-0.016	0.013	0.043	-0.173	0.137	0.463
			rock cover	-0.432	-0.125	0.214	-8.331	-2.412	4.124	-0.225	-0.065	0.111
			shrub cover	-1.344	-0.873	-0.456	-32.664	-21.214	-11.074	-0.553	-0.359	-0.188
			bryophyte cover	-0.878	-0.585	-0.255	-13.164	-8.774	-3.826	-0.586	-0.391	-0.170
			lichen cover	-0.738	-0.446	-0.177	-8.506	-5.140	-2.037	-0.640	-0.387	-0.153
			vegetation height	-0.738	-0.372	-0.013	-2.318	-1.168	-0.041	-0.235	-0.119	-0.004
soil - canopy T difference	200	min	Intercept	-7.611	-7.366	-7.117						
			elevation	-0.130	0.214	0.548	-30.959	50.888	130.029	-0.055	0.090	0.231
			slope angle	-0.447	-0.111	0.232	-3.318	-0.828	1.725	-0.060	-0.015	0.031
			solar radiation	0.068	0.337	0.620	0.004	0.018	0.034	0.013	0.062	0.115
			landform	-0.146	0.121	0.365	-0.126	0.105	0.317	-0.168	0.139	0.421
			soil moisture	0.081	0.376	0.690	0.008	0.036	0.067	0.084	0.389	0.714
			rock cover	-0.254	0.067	0.377	-4.894	1.295	7.264	-0.132	0.035	0.196
			shrub cover	-0.953	-0.531	-0.107	-23.157	-12.910	-2.597	-0.392	-0.219	-0.044
			bryophyte cover	-0.802	-0.530	-0.222	-12.025	-7.940	-3.323	-0.535	-0.353	-0.148

model response	circle radius	temp. variable	predictor	post. 2.5% quantile	post. mean	post. 97.5% quantile	post. 2.5% quantile (backscaled)	post. mean (backscaled)	post. 97.5% quantile (backscaled)	post. 2.5% quantile (°C per unit)	post. mean (°C per unit)	post. 97.5% quantile (°C per unit)
soil - canopy T difference (cont.)	200	min	lichen cover	-0.461	-0.182	0.084	-5.314	-2.098	0.965	-0.400	-0.158	0.073
			vegetation height	-1.188	-0.838	-0.495	-3.728	-2.629	-1.553	-0.378	-0.267	-0.158
soil - canopy T difference	200	max	Intercept	-2.595	-2.181	-1.825						
			elevation	0.107	0.648	1.178	25.496	153.868	279.619	0.045	0.273	0.496
			slope angle	-0.426	0.143	0.682	-3.164	1.062	5.069	-0.057	0.019	0.092
			solar radiation	0.247	0.678	1.146	0.013	0.037	0.062	0.046	0.125	0.212
			landform	0.007	0.425	0.799	0.006	0.369	0.694	0.008	0.490	0.920
			soil moisture	-0.451	0.015	0.496	-0.044	0.001	0.048	-0.467	0.016	0.514
			rock cover	-0.798	-0.292	0.247	-15.374	-5.624	4.766	-0.414	-0.152	0.128
			shrub cover	-2.116	-1.463	-0.789	-51.405	-35.537	-19.166	-0.871	-0.602	-0.325
			bryophyte cover	-1.108	-0.634	-0.185	-16.600	-9.505	-2.769	-0.739	-0.423	-0.123
			lichen cover	-1.195	-0.731	-0.293	-13.774	-8.428	-3.378	-1.037	-0.634	-0.254
			vegetation height	-1.401	-0.864	-0.306	-4.397	-2.711	-0.962	-0.446	-0.275	-0.098
soil - canopy T difference	564	mean	Intercept	0.221	0.498	0.779						
			elevation	0.034	0.411	0.795	8.136	97.523	188.762	0.014	0.173	0.335
			slope angle	-0.275	0.109	0.483	-2.046	0.810	3.590	-0.037	0.015	0.065
			solar radiation	0.049	0.391	0.709	0.003	0.021	0.038	0.009	0.072	0.131
			landform	-0.018	0.258	0.559	-0.016	0.224	0.485	-0.021	0.297	0.644
			soil moisture	-0.236	0.115	0.437	-0.023	0.011	0.042	-0.244	0.119	0.452
			rock cover	-0.314	0.045	0.414	-6.344	0.913	8.372	-0.155	0.022	0.204
			shrub cover	-0.991	-0.516	-0.001	-23.804	-12.386	-0.032	-0.413	-0.215	-0.001
			bryophyte cover	-1.056	-0.741	-0.378	-16.200	-11.376	-5.805	-0.688	-0.483	-0.247
			lichen cover	-0.767	-0.432	-0.108	-9.862	-5.555	-1.393	-0.597	-0.336	-0.084
			vegetation height	-0.769	-0.399	-0.001	-2.572	-1.334	-0.005	-0.230	-0.119	0.000
soil - canopy T difference	564	min	Intercept	-7.647	-7.365	-7.100						
			elevation	-0.069	0.302	0.668	-16.481	71.711	158.611	-0.029	0.127	0.282
			slope angle	-0.690	-0.317	0.039	-5.127	-2.354	0.290	-0.093	-0.043	0.005
			solar radiation	0.086	0.418	0.730	0.005	0.023	0.040	0.016	0.077	0.135
			landform	-0.025	0.260	0.550	-0.021	0.226	0.478	-0.028	0.300	0.634
			soil moisture	0.086	0.406	0.733	0.008	0.039	0.071	0.089	0.420	0.759
			rock cover	-0.130	0.216	0.589	-2.622	4.377	11.928	-0.064	0.107	0.291

model response	circle radius	temp. variable	predictor	post. 2.5% quantile	post. mean	post. 97.5% quantile	post. 2.5% quantile (backscaled)	post. mean (backscaled)	post. 97.5% quantile (backscaled)	post. 2.5% quantile (°C per unit)	post. mean (°C per unit)	post. 97.5% quantile (°C per unit)
soil - canopy T difference (cont.)	564	min	shrub cover	-0.604	-0.110	0.380	-14.508	-2.643	9.131	-0.252	-0.046	0.158
			bryophyte cover	-0.955	-0.640	-0.320	-14.658	-9.817	-4.917	-0.623	-0.417	-0.209
			lichen cover	-0.540	-0.219	0.106	-6.947	-2.816	1.357	-0.420	-0.170	0.082
			vegetation height	-1.320	-0.943	-0.568	-4.414	-3.155	-1.899	-0.395	-0.282	-0.170
soil - canopy T difference	564	max	Intercept	-2.598	-2.157	-1.723						
			elevation	0.129	0.737	1.367	30.598	174.886	324.492	0.054	0.310	0.576
			slope angle	-0.674	-0.065	0.592	-5.004	-0.484	4.397	-0.091	-0.009	0.080
			solar radiation	0.140	0.650	1.219	0.008	0.035	0.066	0.026	0.120	0.225
			landform	0.101	0.576	1.108	0.088	0.500	0.962	0.116	0.663	1.276
			soil moisture	-0.571	-0.014	0.576	-0.055	-0.001	0.056	-0.591	-0.014	0.596
			rock cover	-0.622	-0.033	0.554	-12.595	-0.670	11.215	-0.308	-0.016	0.274
			shrub cover	-2.003	-1.114	-0.350	-48.115	-26.748	-8.401	-0.834	-0.464	-0.146
			bryophyte cover	-1.231	-0.705	-0.113	-18.890	-10.812	-1.738	-0.802	-0.459	-0.074
			lichen cover	-1.258	-0.714	-0.182	-16.172	-9.178	-2.337	-0.978	-0.555	-0.141
			vegetation height	-1.357	-0.737	-0.140	-4.540	-2.466	-0.470	-0.406	-0.220	-0.042

Table S7: Predicted threshold cover percentages for switch from warmer to colder soils compared to canopy-level mean temperatures during the growing season. See also Figure 4 a,d,g for graphed relationships.

sample plot size (radius)	cover variable	threshold cover %
small (0.50m)	shrubs	50.99
small (0.50m)	bryophytes	29.06
medium (2.00m)	shrubs	44.87
medium (2.00m)	bryophytes	23.21
large (5.64m)	shrubs	56.44
large (5.64m)	bryophytes	22.50

Table S8: Pearson correlation coefficients for shrub and bryophyte cover and average vegetation height across sample plot sizes.

variable	bryophyte cover (r = 0.5 m)	bryophyte cover (r = 2.0 m)	bryophyte cover (r = 5.64 m)	shrub cover (r = 0.5 m)	shrub cover (r = 2.0 m)	shrub cover (r = 5.64 m)	average vegetation height (r = 0.5 m)	average vegetation height (r = 2.0 m)	average vegetation height (r = 5.64 m)
bryophyte cover (r = 0.5 m)	1.00	0.84	0.78						
bryophyte cover (r = 2.0 m)	0.84	1.00	0.89						
bryophyte cover (r = 5.64 m)	0.78	0.89	1.00						
shrub cover (r = 0.5 m)				1.00	0.86	0.79			
shrub cover (r = 2.0 m)				0.86	1.00	0.89			
shrub cover (r = 5.64 m)				0.79	0.89	1.00			
average vegetation height (r = 0.5 m)							1.00	0.90	0.63
average vegetation height (r = 2.0 m)							0.90	1.00	0.83
average vegetation height (r = 5.64 m)							0.63	0.83	1.00
